# Transcriptomic and Functional Evidence for Differential Effects of MCF-7 Breast Cancer Cell-Secretome on Vascular and Lymphatic Endothelial Cell Growth

**DOI:** 10.3390/ijms23137192

**Published:** 2022-06-28

**Authors:** Giovanna Azzarito, Michele Visentin, Brigitte Leeners, Raghvendra K. Dubey

**Affiliations:** 1 Department of Reproductive Endocrinology, University Hospital Zurich, 8952 Schlieren, Switzerland; giovanna.azzarito@usz.ch (G.A.); brigitte.leeners@usz.ch (B.L.); 2 Department of Clinical Pharmacology and Toxicology, University Hospital Zurich, University of Zurich, 8091 Zurich, Switzerland; michele.visentin@usz.ch; 3 Department of Pharmacology & Chemical Biology, University of Pittsburgh, Pittsburgh, PA 15219, USA

**Keywords:** VECs, LECs, secretome, metabolism, immune, angiogenesis, lymphangiogenesis, proliferation, breast cancer

## Abstract

Vascular and lymphatic vessels drive breast cancer (BC) growth and metastasis. We assessed the cell growth (proliferation, migration, and capillary formation), gene-, and protein-expression profiles of Vascular Endothelial Cells (VECs) and Lymphatic Endothelial Cells (LECs) exposed to a conditioned medium (CM) from estrogen receptor-positive BC cells (MCF-7) in the presence or absence of Estradiol. We demonstrated that MCF-7-CM stimulated growth and capillary formation in VECs but inhibited LEC growth. Consistently, MCF-7-CM induced ERK1/2 and Akt phosphorylation in VECs and inhibited them in LECs. Gene expression analysis revealed that the LECs were overall (≈10-fold) more sensitive to MCF-7-CM exposure than VECs. Growth/angiogenesis and cell cycle pathways were upregulated in VECs but downregulated in LECs. An angiogenesis proteome array confirmed the upregulation of 23 pro-angiogenesis proteins in VECs. In LECs, the expression of genes related to ATP synthesis and the ATP content were reduced by MCF-7-CM, whereas MTHFD2 gene, involved in folate metabolism and immune evasion, was upregulated. The contrasting effect of MCF-7-CM on the growth of VECs and LECs was reversed by inhibiting the TGF-β signaling pathway. The effect of MCF-7-CM on VEC growth was also reversed by inhibiting the VEGF signaling pathway. In conclusion, BC secretome may facilitate cancer cell survival and tumor growth by simultaneously promoting vascular angiogenesis and inhibiting lymphatic growth. The differential effects of BC secretome on LECs and VECs may be of pathophysiological relevance in BC.

## 1. Introduction

Breast cancer (BC) is the most diagnosed and malignant cancer worldwide and a leading cause of mortality in women [1,2]. Cellular and molecular investigations provide evidence that tumors are dynamic tissue containing multiple cell types (breast cancer epithelial cells, fibroblasts/stromal cells, blood vessels, lymphatic vessels, and immune cells) that interact via locally generated paracrine factors [3,4]. It is postulated that factors within the tumor milieu promote vascular angiogenesis and lymphangiogenesis and support tumor growth, progression, and metastasis. Moreover, pharmacological molecules inhibiting neovascularization are effective in treating tumor growth [5,6]. 

The role of neo-angiogenesis in supporting tumor growth is well-established [7,8,9]. During tumorigenesis, an increase in new vessel formation from pre-existing vessels (angiogenesis) supplies oxygen and nutrients to the tumor, thereby promoting its growth [8,10,11,12,13,14]. Compared with vascular capillaries, the functional role of lymphatic vessels in BC/tumors is less clear. Lymphatic endothelial cells (LECs) originate from a subpopulation of vascular endothelial cells (VECs) in the cardinal vein or from LEC progenitors in the intersegmental vessels during embryogenesis [7]. Importantly, their structure and functional characteristics vary considerably from VECs [7]. The increased lymphatic vessel density in tumors is largely due to growth of pre-existing conduits [15,16]; however, some tumor-associated LECs are documented to be of non-venous origin and of mesenchymal origin [17,18]. Unlike the blood vessels, discrepant findings with regard to increased lymphatic vessel density and tumor growth/metastasis have been reported [17,18].

The lymphatic vessels serve as a conduit to channel immune cells to counteract cancer cell growth [19]. Indeed, crosstalk between lymphatic and immune cells plays an important role in the uptake/migration of cells into the lymphatics [20] via LEC-generated chemokine(s) gradients [21]. Additionally, lymphatic vessels serve as a channel to spread cancer cells from their place of origin to the regional lymph nodes [22]. Active lymphangiogenesis occurs during embryogenesis but is absent in adulthood. However, lymphangiogenesis can be induced under pathological conditions such as cancer and inflammation [23,24]. Hence, a deeper understanding of the role of lymphangiogenesis and angiogenesis in BC growth and spread is critical in the development of therapeutic approaches.

Several endogeous factors that promote or inhibit angiogenesis have been identified in BC, including TGF-β (transforming growth factor-β) and VEGF (vascular endothelial growth factor). TGF-β, a biphasic growth regulator, plays an important role in both vascular and lymphatic endothelial cells (EC). In VECs, TGF-β stimulates angiogenesis by inducing pro-angiogenic factors [14]. Several studies have identified the overexpression of TGF-β in various types of human cancers/tumors, including BC, and this correlates with tumor progression and poor prognostic outcome [25]. Interestingly, TGF-β negatively regulates lymphangiogenesis in certain cancer tissues and inhibits LECs’ proliferation, cord formation, and migration through the repression of LEC-related genes [14,15]. Apart from TGF-β, other cytokines and growth factors also influence vascular and lymphatic EC growth in a differential fashion. For example, VEGF-A induces growth of vascular endothelial cells via VEGF-R2 but not in LECs [26], whereas VEGF-C induces growth in LECs via VEGF-R3 [27,28,29].

The molecules triggering EC’s growth and angiogenesis largely mediate their actions by activating mitogen-activated protein kinases (MAPK or ERK1/2) and/or phosphatidylinositol-3-kinase (PI3K)/Akt. Although the roles of TGF-β, VEGF, MAPK, and Akt in regulating neo-angiogenesis have been investigated, their relative role in mediating the effects of paracrine factors generated by cancer cells/BCs in LECs and VECs is unclear. Since cancer cells generate multiple factors, the effects of a conditioned medium (CM) on MAPK and Akt activity would better reflect the effects of cancer cell secretome on VEC and LEC activity.

Neo-angiogenesis in tumors/BC is partly driven by paracrine factors, generated by cancer cells. Whether BC-derived soluble/paracrine factors induce similar actions on vascular and lymphatic EC growth is unclear. Moreover, the impact of BC-derived paracrine factors on VEC and LEC genes remains poorly documented. Since discrepant findings with regard to role of lymphatic vessels in metastasis have been reported, together with the fact that the lymphatic system acts as an immune sink to cleanse the cancer cells [19,22] and intra-tumoral lymphatic vessels appear collapsed and dysfunctional [17], it is important to study the effects of BC-driven changes in LECs and VECs. Apart from signal transduction pathways (MAPK and Akt), EC metabolism and mitochondrial activity have also emerged as key players in regulating vascular and lymphatic angiogenesis [30,31,32,33,34]. Hence, a better understanding of the impact of BC cell-derived paracrine factors on VEC and LEC growth at functional and molecular level is needed.

CM from tumors, LECs, and VECs have been used to study the effects of tumor cells on cancer cell growth and metastasis [35]. However, much less is known about the influence of factors generated by cancer cells on lymphatic and vascular EC growth (proliferation, migration, and capillary formation). Hence, the goal of this study was to simultaneously determine and compare the effects of MCF-7-derived soluble factors or CM on LEC and VEC growth (proliferation, migration, and angiogenesis), gene, and protein expression, as well as signal transduction pathways. Since estradiol (E2) is known to promote MCF-7 cell growth, we also investigated the impact of CM obtained from MCF-7 cells pre-treated with and without E2 on the growth of LECs and VECs.

## 2. Results

### 2.1. Effect of Estrogen and CM on VEC Proliferation

**Figure 1:**
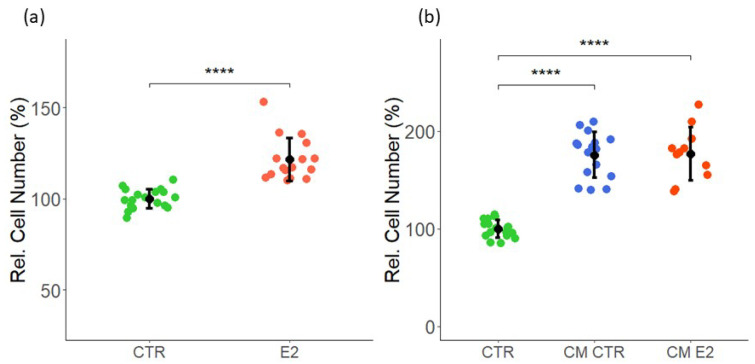
**Effects of E2 and CM from MCF-7 cells treated with or without E2 on VEC proliferation**. (**a**) VECs were treated with 10 nM of E2 and its vehicle (DMSO) and (**b**) cultured in CMs for 48 h. The CM CTR and CM E2 were formed by MCF-7 cells pre-treated with E2 and DMSO (vehicle) for 24 h. The cell proliferation was assessed by cell counting. (**a**) CTR = 10 nM DMSO (control), E2 = 10 nM E2; (**b**) CTR = serum-free medium not added to cells, CM CTR/E2 = E2/vehicle (DMSO) treatment on MCF-7 for 24 h replaced with serum-free medium, and after 48 h, the supernatant was collected. The experiments were performed at least three times in triplicates or quadruplicates, and values are expressed as mean ± SD. **** *p* < 0.001 compared to the respective control.

Consistent with the established pro-mitogenic actions of estrogen, the treatment of MCF-7 cells with estradiol induced cell proliferation [36,37] (Supplementary Data S1). Moreover, the treatment of VECs with estradiol for 3 days increased proliferation by 22 ± 12 (*p* < 0.05 vs. untreated control), as assessed by the change in cell number (Figure 1a). To assess whether MCF-7 secretome modulated VEC growth, we collected a conditioned medium (CM) from cultured MCF-7 cells pre-treated with and without E2 (CM E2 and CM CTR). Moreover, the medium collected in absence of MCF-7 cells served as a control (CTR). Compared with the control, the treatment of VECs with CM from MCF-7 cells treated with or without E2 significantly induced proliferation by 76 ± 23 and 77 ± 27 (*p* < 0.05 vs. untreated control), respectively (Figure 1b). Although E2 alone induced MCF-7 proliferation, it did not enhance the mitogenic effects of MCF-7 CM further. These observations suggest that soluble factors secreted by MCF-7 cells can induce VECs’ proliferation.

### 2.2. Effect of Estrogen and CM on LEC Proliferation

**Figure 2:**
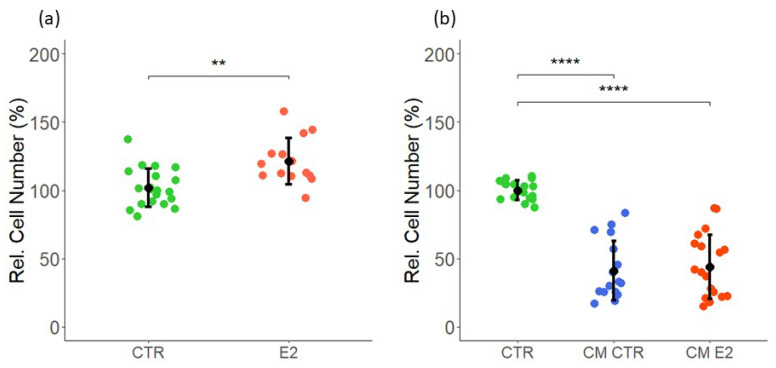
**Effects of E2 and CM from MCF-7 cells treated with or without E2 on LEC proliferation**. (**a**) LECs were treated with 10 nM E2 or DMSO (vehicle) as the control and (**b**) cultured in CM for 48 h, and cell proliferation was assessed by cell counting. CM CTR and CM E2 were formed by MCF-7 cells pre-treated with E2 and DMSO (vehicle). (**a**) CTR = 10 nM DMSO (control), E2 = 10 nM E2; (**b**) CTR = serum-free medium not added to cells, CM CTR/E2 = E2/vehicle (DMSO) pre-treatment on MCF-7. Experiments were performed at least three times in triplicates or quadruplicates, and the data represent mean ± SD. **** *p* < 0.0001, ** *p* < 0.001 compared to the respective control.

Similar to VECs, we assessed the effects of E2 on LEC proliferation. As shown in Figure 2a, the treatment of LECs with 10 nM E2 for 3 days induced cell proliferation by 21 ± 17 (*p* < 0.05 vs. untreated control). Next, similar to VECs, we assessed the impact of MCF-7 CM on LEC growth following treatment for 48 h. In contrast to VECs, CM from MCF-7 cells treated in presence or absence of E2 strongly inhibited LEC proliferation, as assessed by cell counting. Compared with the LECs treated with the control medium (CTR), the CM from MCF-7 cells exposed in the absence (CM CTR) or presence of E2 (CM E2) inhibited LEC growth by 60 ± 22 and 56 ± 23 (*p* < 0.05 vs. untreated control), respectively (Figure 2b). Taken together, our findings provide evidence that MCF-7 secretome contains soluble factors, which differentially influence VEC and LEC proliferation.

### 2.3. CM Exhibits Angiogenic Potential in Tube Formation Assay

**Figure 3:**
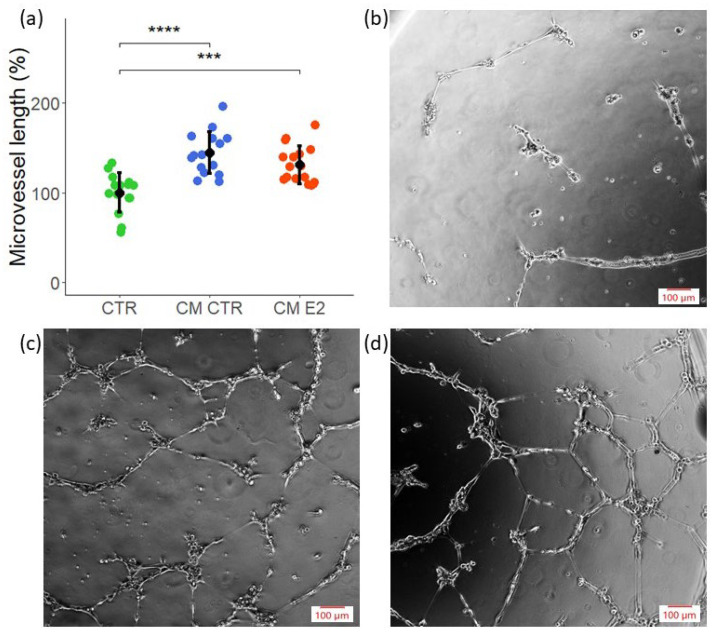
**CM from MCF-7 cells promotes vasculogenesis**. Tube formation by VECs was investigated using Matrigel-based assay. Cells were incubated for 30 min with CM in 0.4% FCS before plating on Matrigel. Cells were allowed to form tube-like structures for 16–18 h. (**a**) Tube length was measured microscopically compared with the respective control. Experiments were performed at least three times in triplicates or quadruplicates, and the values are expressed as mean ± SD, **** *p* < 0.001, *** *p* < 0.005. (**b**–**d**) Photomicrographs depict representative images for each condition: CTR, CM CTR, and CM E2. Scale bar, 100 µm.

Angiogenesis facilitates invasion, growth, and progression in a variety of tumors. Hence, we assessed the impact of MCF-7 CM on angiogenesis by studying capillary formation. Unlike the VECs, the LECs failed to form capillaries under identical conditions. Hence, we only assessed the impact of CM on capillary formation by VECs. As shown in Figure 3a, MCF-7 CM induced micro-vessel formation. Moreover, comparable stimulatory effects were observed in response to the MCF-7 CM collected in E2-exposed cells (CM E2). Representative photomicrographs in Figure 3b–d depict capillary formation and branching in VECs exposed to control medium (CTR), CM from MCF-7 cells, and CM from MCF-7 treated in presence of E2 (Figure 3b–d). These results demonstrate that MCF-7 secretome induces capillary formation by VECs.

### 2.4. Effect of Conditioned Media on Cell Migration

**Figure 4:**
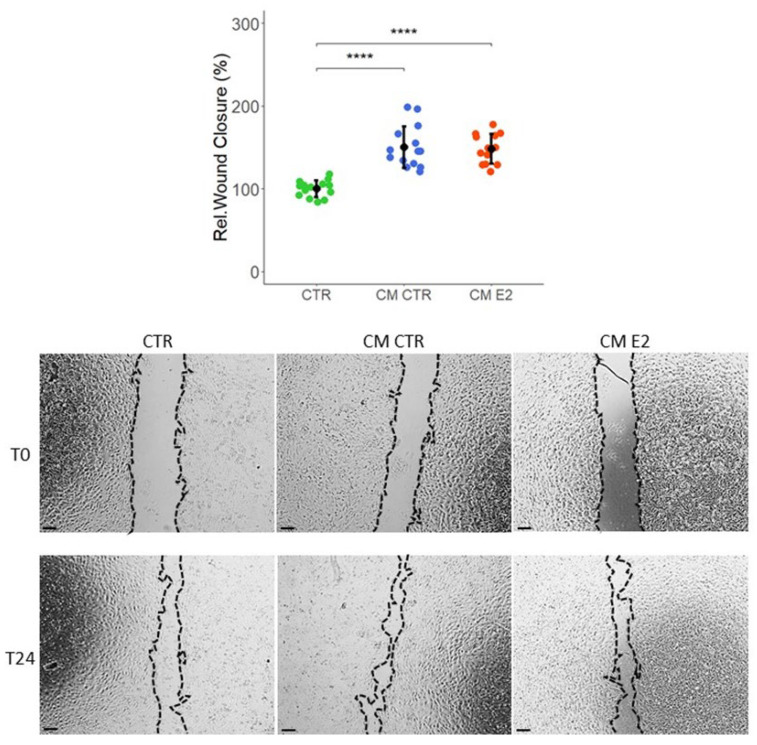
**MCF-7-derived CM stimulates VEC migration**. Cell migration was investigated by a wound closure assay in the confluent VECs’ monolayer. Cells were cultured in CM CTR/E2 in 0.4% FCS after the scratch was induced. Representative images are shown immediately after the scratch (T0) and at 24 h (T24) for each condition. Experiments were performed at least three times in triplicates or quadruplicates. **** *p* < 0.001, compared with the respective control. Values are expressed as mean ± SD. Scale bar, 200 µm.

Cell migration regulation is vital to angiogenesis and metastasis. To further investigate the effects of conditioned medium on migration of VECs, we performed a wound-healing scratch assay in the presence of CM collected from MCF-7 cells treated with or without E2. As shown in Figure 4, the CMs collected from MCF-7 cells treated with or without E2 increased the migration of VECs by 48 ± 18 and 50 ± 25 (CM CTR and CM E2, respectively). Our results from angiogenesis and scratch-wound assays show that MCF-derived factors in CM induce VEC migration, and these effects are not further enhanced by E2.

### 2.5. Role of MAPK (ERK1/2) and PI3K-Akt Pathways in Mediating the Effects of CM on VEC and LEC Proliferation

**Figure 5:**
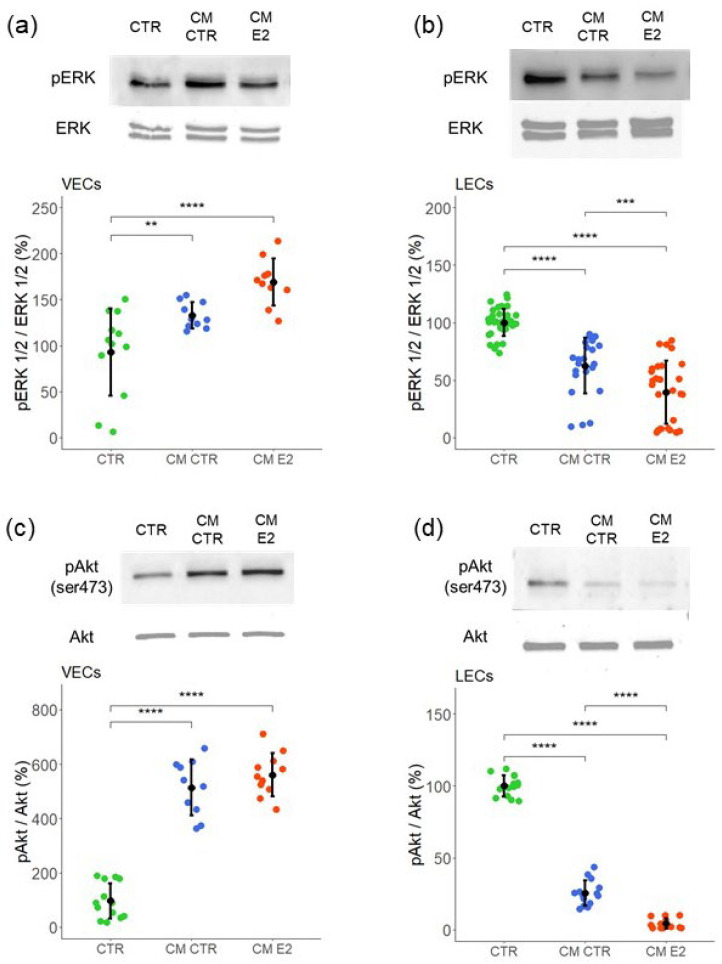
**MCF-7-derived CM induces ERK 1/2 and AKT kinase phosphorylation in VECs (panels (a,c)) and inhibits their activity in LECs (panels (b,d)).** Representative western blots and graphs for ERK 1/2 and AKT phosphorylation after culturing in MCF-7-CM for 45 min. Total ERK and total AKT were used as loading control. Experiments were performed at least three times in triplicates or quadruplicates, and the values are expressed as mean ± SD. **** *p* < 0.001, *** *p* < 0.01, ** *p* < 0.05 compared with the respective control.

To determine the signal transduction pathways involved in mediating CM action in VECs and LECs, a western blot analysis was performed on the whole cell lysate. Mitogen-activated protein kinases’ (MAPK) signaling pathway is one of the most important for cell proliferation, migration, and cell survival. An aberrant activation of this pathway is a major oncogenic event in many human cancers [38]. To investigate the role of ERK-MAPK in VECs and LECs, we examined whether MCF-7 CM modulated ERK 1/2 phosphorylation. The treatment of VECs to CM CTR and CM E2 increased ERK 1/2 phosphorylation by 33 ± 14 and 70 ± 25.5, respectively (Figure 5a). In contrast with VECs, the treatment of LECs with CM CTR and CM E2 inhibited ERK 1/2 phosphorylation by 37 ± 24 (*p* < 0.05 vs. untreated control) and 60 ± 30 (*p* < 0.05 vs. untreated control) (Figure 5b).

In addition to MAPK, Akt (Protein kinase B) activation/phosphorylation actively regulates cell survival, cell growth, and migration. The PI3K–Akt signaling pathway is a pro-angiogenic pathway and is active in several cancers [39,40]. To assess whether MCF-7 secretome in CM modulated PI3K/AKT signaling in VECs and LECs, individual cell monolayers were exposed to MCF-7 CM, and Akt phosphorylation was assessed by western blotting. In VECs, treatment with MCF-7 CM (CM CTR) as well as CM from E2-exposed MCF-7 cells (CM E2) significantly induced Akt phosphorylation by 413 ± 103 and 460 ± 80 (*p* < 0.05 vs. untreated control), respectively (Figure 5c). In contrast to VECs, treatment of LECs with CM CTR and CM E2 inhibited Akt phosphorylation by 74 ± 9 and 95 ± 4 (*p* < 0.05 vs. untreated control), respectively. Interestingly, the inhibitory effects of CM collected from MCF-7 cells in the presence of E2 were significantly higher (Figure 5d). Taken together, our results show that the stimulatory effects of MCF-7 CM on VEC growth are accompanied by MAPK and Akt activation, whereas the inhibitory effects of MCF-7 CM on LEC growth are accompanied by MAPK and Akt inhibition. Hence, both these pathways may play a key role in regulating the effects of the tumor micro-environment on angiogenesis and lymphangiogenesis.

### 2.6. Microarray Analysis

#### 2.6.1. Differentially Regulated Genes in VECs Cultured in CM

**Figure 6:**
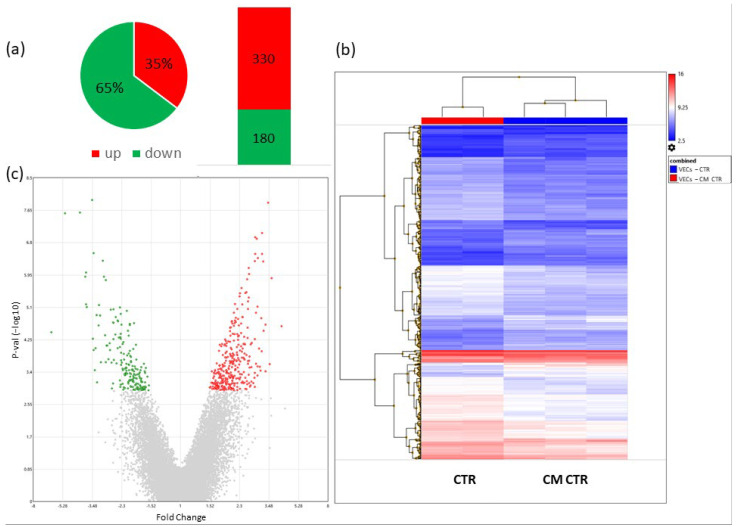
**Differentially regulated genes (DRGs) in VECs cultured in MCF-derived CM.** Number of DRGs and pie chart representation of up- and downregulated genes in percentage of total number of DRGs (**a**). Heatmap representation of DRGs between CM CTR vs. CTR (**b**). Volcano plot showing the most upregulated genes (red), the most downregulated genes (green), and the most statistically significant genes are toward the top (**c**). Transcriptome Analysis Console (TAC, Applied Biosystems) was used for analyzing the gene expression data.

To determine the potential genes involved in mediating the effects of MCF-7 CM on VEC proliferation, migration, and angiogenesis, we performed microarray analysis. The differentially regulated genes (DRGs) were determined by the comparison of gene expression in VECs treated with CM vs. the relative control. A change of 1.5-fold and *p*-value < 0.05 served as cut-off criteria, a total of 510 DRGs were identified that contained 330 upregulated DRGs and 180 downregulated DRGs (Figure 6).

A list of the top ten genes up- and downregulated in VECs cultured in CM is shown in Table 1 and Table 2, respectively.

##### Validation of Regulated Genes by Real-Time PCR

**Figure 7:**
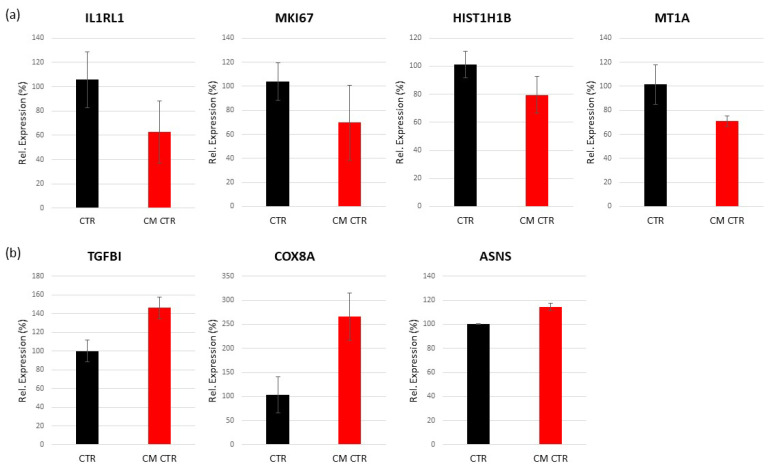
**Validation of genes in VECs.** Seven genes were selected to be validated by Custom RT^2^ Profiler PCR Array from Qiagen. ILRL1, MKI67, HIST1H1B, and TGFBI were the highly regulated genes, and MT1A, COX8A, and ASNS were randomly chosen. For data normalization, GAPDH and LDHA were used as internal controls. Down- (**a**) and upregulated genes (**b**) were consistent with the microarray results. The experiment was performed in triplicates.

To identify the bioinformatics analysis associated with the regulation of the highly regulated genes in VECs, we selected the TGFBI, COX8A, and ASNS genes (upregulated) and IL1RL1, MKI67, HIST1H1B, and MT1A genes (downregulated) for RT-PCR verification. The results show that the relative expression levels of the genes were consistent with the microarray hybridization for both up- and downregulated genes (Figure 7).

#### 2.6.2. Differentially Regulated Genes in LECs Cultured in CM

**Figure 8:**
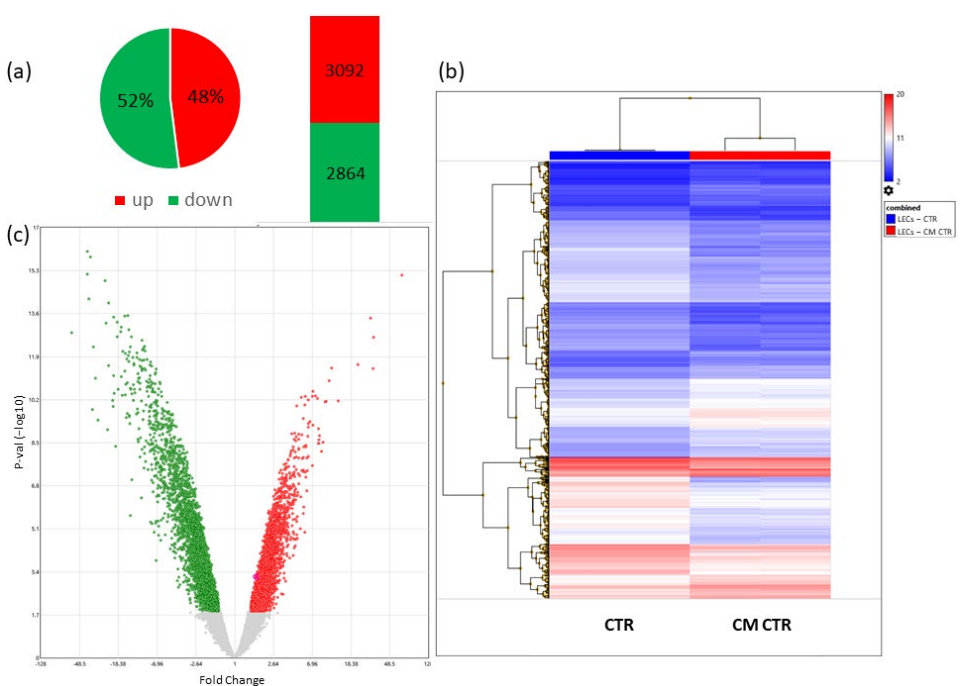
**Differentially regulated genes (DRGs) in LECs cultured in CM.** Number of DRGs and pie chart representation of up- and downregulated genes in percentage of total number of DRGs (**a**). Heatmap representation of DRGs between CM CTR vs. CTR (**b**). Volcano plot showing the most up-regulated genes (red), the most down-regulated genes (green), and the most statistically significant genes are towards the top (**c**). Transcriptome Analysis Console (TAC, Applied Biosystems) was used for analyzing gene expression data.

In order to identify the genes regulated by CM in LECs, we performed a microarray analysis. Using a cut off *p*-value of < 0.05 and 1.5-fold change (FDR), we identified 5956 differentially regulated genes (DRGs) in LECs cultured in CM compared with LECs cultured in the control medium. Furthermore, the volcano plot showed that among these DEGs, 2864 genes were upregulated, and 3092 genes were downregulated (Figure 8).

The top ten up- and downregulated genes are listed in Table 3 and Table 4, respectively.

##### Validation of Regulated Genes by Real-Time PCR

**Figure 9:**
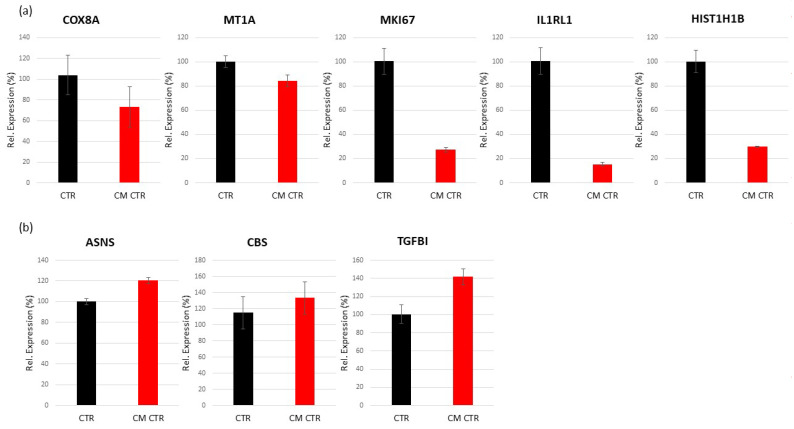
**Validation of gene in LECs.** Eight genes were selected to be validated by Custom RT^2^ Profiler PCR Array from Qiagen. COX8A, MT1A, ASNS, and CBS are the highly regulated genes, and MKI67, IL1RL1, HIST1H1B, and TGFBI were randomly selected. For data normalization, GAPDH and LDHA were used as internal controls. Down- (**a**) and upregulated genes (**b**) were consistent with the microarray results. The experiment was performed in triplicates.

To validate the results of the microarray, a real-time PCR was carried out using Custom RT^2^ Profiler PCR Array. We selected a total of eight genes: MKI67, IL1RL1, HIST1H1B, and TGFBI were randomly chosen, and COX8A, MT1A, ASNS, and CBS are highly expressed genes. As a result, the expression differences obtained by RT-PCR were consistent with the results of the transcriptional analysis: ASNS, CBS, and TGFBI showed a nice increase in PCR, while COX8A, MT1A, MKI67, IL1RL1, and HIST1H1B showed an inhibition of gene expression in the LECs cultured in CM compared with the control (Figure 9). The relative expression of each gene was calculated according to the relative expression quantity  =  2^−ΔCT^ formula, where ΔCT  =  CT value of target gene–CT value of internal reference gene (GAPDH and LDHA).

#### 2.6.3. Pathway Enrichment Analysis of DRGs in VECs

To elucidate the biological functions of the DRGs in VECs cultured in CM vs. its control, regulated genes were mapped to the Enrichr (interactive resource for analyzing gene sets). We screened significantly enriched GO terms, Bioplanet, and KEGG pathways using FDR < 0.05. A biological pathway enrichment analysis found that the DRGs were mainly enriched in cell cycle pathways such as Polo-like kinase 1 (PLK1) pathways, Aurora B signaling, M phase pathways, p53 activity regulation, Cyclin A/B1-associated events during G2/M transition, phosphorylation of Emi1, FOXM1 transcription factor network, mitotic prometaphase, and cytokinesis. Moreover, Lysosome, systemic lupus erythematosus, miRNA regulation of DNA damage response, kinesis, and integrative BC pathways were also regulated (Table 5).

##### Genes’ Identification in Biological Pathways

The cell-cycle-regulated genes were identified by a microarray analysis of endothelial CM-cultured cells. The cell-cycle process was associated with the expression of a set of genes, such as histone cluster 1 H2A/H2B/H3 family members (HIST1H2AG, HIST1H2AJ, HIST1H2AK, HIST1H2AL, HIST1H2AM, HIST1H2BI, HIST1H2BK, HIST1H2BN, HIST1H3J, and HIST1H2BF), kinesin family members (KIF11, KIF15, KIF20A, KIF23, and KIF4A), some Cyclins (CCNA2, CCNB2, CCND2, and CDK1), casein kinases (CSNK1D and CSNK1G1), and Tyrosine-protein kinase/Serine/Threonine Kinase 1 (ABL and AKT1), as well as Mucolipin 1 (MCOLN1), kinetochore complex (NDC80), Sirtuin 1 (SIR1), Sortilin 1 (SORT1), tubulins (TUBA4A and TUBGCP4), and minichromosome maintenance complex (MCM7, MCM9). The complete list is shown in Table 6.

#### 2.6.4. Pathway Enrichment Analysis of DRGs in LECs

To investigate the biological functions of the differentially expressed genes, we up-loaded the 5956 genes to Enrichr. We employed Bio-Planet, GO terms, and KEGG databases for functional enrichment analysis of differentially expressed genes. Enrichr identified 210 pathways in BioPlanet, 80 in KEGG, and 287 in GO biological process with adjusted *p*-value < 0.05. Surprisingly, compared with VECs, the number of DRGs and enriched pathways were dramatically higher in LECs.

The genes involved in these biological processes mainly encode proteins that control translation, transcription, gene expression, endocytosis, phagosome, DNA replication, oxidative phosphorylation, and metabolism, as well as mRNA processing and splicing. Additionally, cell cycle regulation is also implicated in the functional enrichment of LECs cultured in CM, such as mitotic phases transition (G1-G1/S phases, G2-G2/M phases, and prometaphase), meiosis, and mitosis, including programmed cell death and apoptosis regulation. The full list of pathways following pathway enrichment analysis under BioPlanet, KEGG, and GO biological processes are shown in Supplementary Table S1, whereas a list of significantly regulated pathways that were common between Bioplanet and/or KEGG and GO biological processes is shown in Table 7.

##### Genes’ Identification in Biological Pathways

These signaling pathways in LECs have several differentially expressed genes, such as members of the RPL and RPS (Ribosomal protein genes) families, EIF (eukaryotic initiation factors gene family, EIF-1 to EIF-5), PSM (proteasome subunit alpha PSMA, beta PSMB, PSMC, PSMD, and proteasome activator PSME), NUP (nucleoporin), NDUF (NADH dehydrogenase), EXOSC (exosome components), MRP (mitochondrial ribosomal protein MRPL, MRPS), HIST1H (histone cluster), COX (cytochrome c oxidase), and SNORD (small nucleolar RNA).

Importantly, genes and pathways associated with oxidative phosphorylation, mitochondrial metabolism, metallothiones, and immune evasion were significantly modulated in LECs but not in VECs as shown in Table 8. The modulation of specific genes by CM was consistent with the differential growth effects of MCF-7 secretome on VECs and LECs. In this context, the M phase, mitotic prometaphase, mitotic spindle organization, and microRNA regulation of DNA damage response in both VECs and LECs. The pro-growth/angiogenic and cell cycle regulatory pathways, such as integrated breast cancer pathway, polo-like kinase-1, p73 transcription factor network, Aurora B signaling p53 activity, kinesins, FOXM1 transcription factor network, lysosomes, and cyclin A/B1-associated events for G2/M transition, dominated in VECs, whereas the S phase, mitotic G1-G1/S and G2/G2/M phases, cyclin A-cdk2-associated events at S phase entry, cell cycle checkpoints, APC/C-mediated degradation of cell cycle proteins, negative regulation of mitotic cell cycle and G2/M phase transition, negative regulation of ubiquitin-dependent protein catabolic process, respiratory electron transport, cytoplasmic ribosomal proteins, protein metabolism, and Ras and Rho proteins on G1 to S transition in LECs.

**Figure 10:**
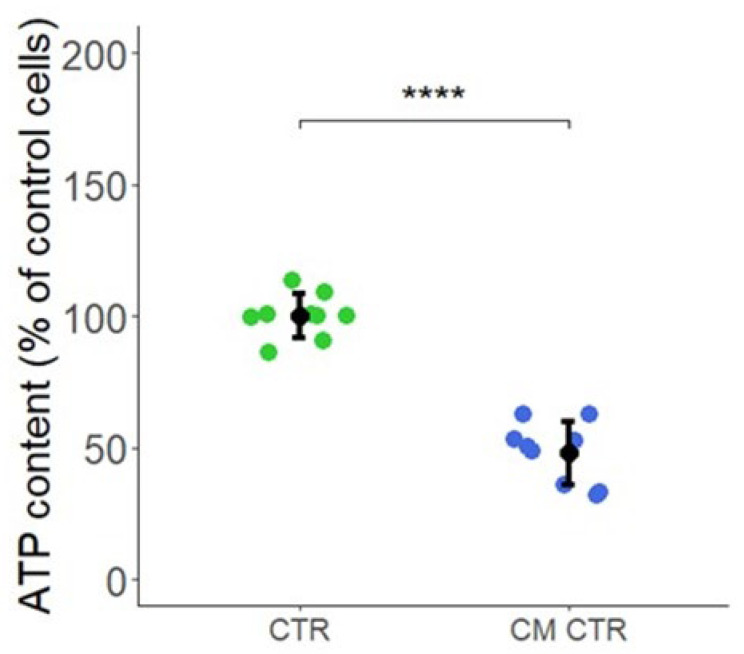
**ATP content is reduced in LECs exposed to MCF-7 CM.** LECs were cultured in CM CTR for 48 h, and ATP levels were measured by luminescent signal. The amounts of ATP were normalized to the relative control. CM CTR was formed by MCF-7 cells pre-treated with DMSO (vehicle). Experiments were performed at least three times in triplicates. **** *p* < 0.0001, compared to the CTR = serum-free medium not added to cells, and CM CTR = vehicle (DMSO) pre-treatment on MCF-7. Values are expressed as mean ± SD.

Among the key pathways that significantly downregulated CM-treated LECs was cellular/mitochondrial metabolism. To confirm the impact of CM on LEC mitochondrial metabolism, we assessed the impact of CM on ATP levels in LECs. As shown in Figure 10, the treatment of LECs with CM significantly reduced ATP formation, confirming that CM downregulated LEC metabolism and inhibited mitochondrial respiration. Microarray data revealed that the genes for asparagine synthetase (ASNS), cystathionine-beta-synthase (CBS), and MTHFD2 that are associated with metabolism were in the top 10 genes upregulated in LECs in response to CM.

### 2.7. Angiogenesis Proteome Profiler in VECs

**Figure 11:**
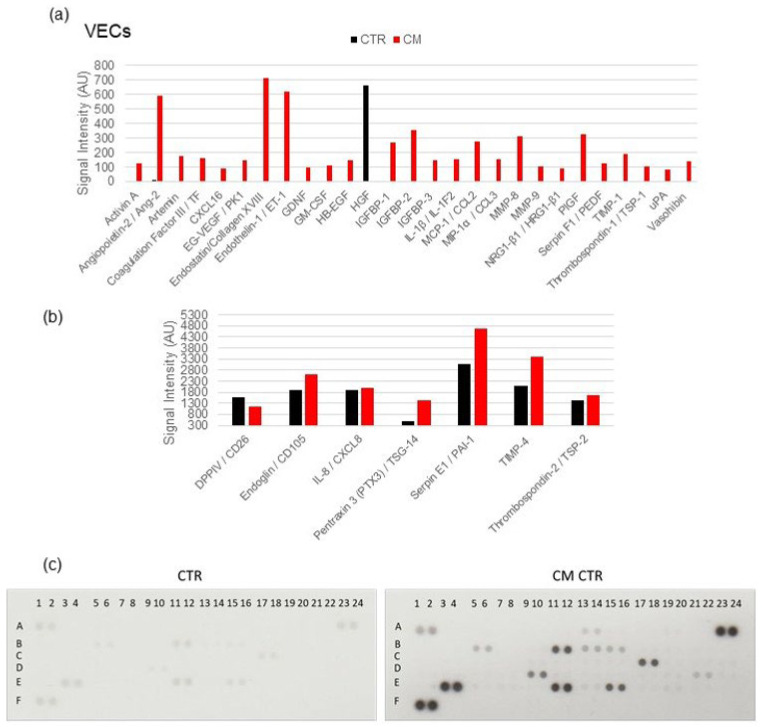
**CM induced the expression of pro-angiogenic proteins in VECs.** An angiogenic protein profile array was performed using 185 µg of protein from VECs cultured for 48 h in CM CTR. Images were analyzed using ImageJ after background subtraction. Bar graphs show the average signal intensities of the framed spots on the array blots and include expression levels changes between 0 and 800 AU (**a**) and between 300 and 5000 AU (**b**). Representative array blots are shown after an exposure time of 5 min (**c**). Experiment was performed twice with independent samples.

**Figure 12:**
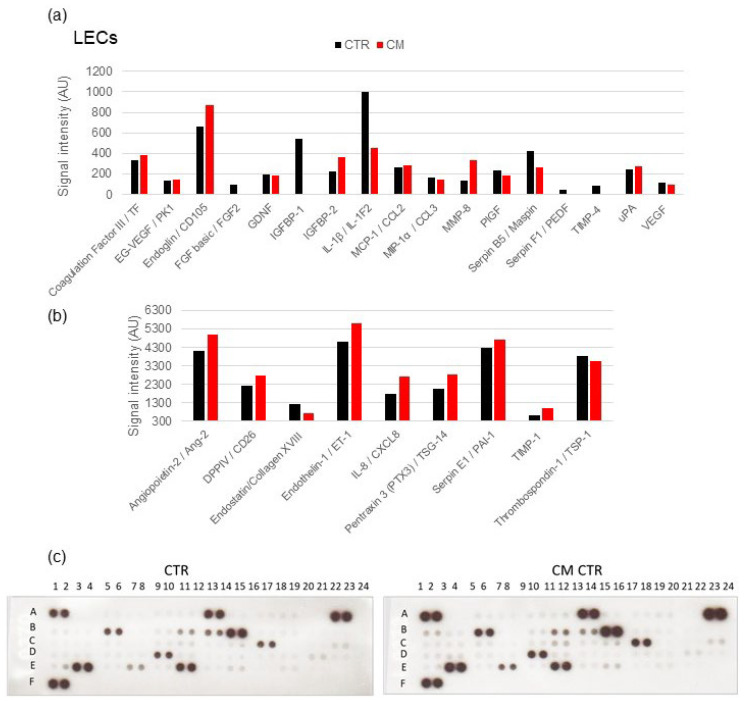
**CM modulated the secretion of pro-angiogenic proteins in LECs.** An angiogenic protein profile array was performed using a Proteome Profiler Human Angiogenesis Array kit. The average pixel density of the pair of duplicate spots were analyzed using ImageJ after background subtraction. We used 190 µg of cell lysates from LECs cultured in CM. Changes in the signal intensity in MCF-7 cells’ secreted proteins are shown in the bar graphs, including expression levels changes between 0 and 1200 AU (**a**) and between 300 and 6000 AU (**b**). Representative array blots are shown after an exposure time of 4 min (**c**). The experiment was performed twice with independent samples.

Since cell proliferation was increased in VECs cultured in CM, we hypothesized that MCF-7 cells would secrete different factors that could differentially affect tumor growth. To test this hypothesis, we used a Proteome Profiler Antibody Arrays Kit for Human Angiogenesis to determine 55 angiogenesis-related factors in CM. To quantify the expression, the means of the pixel number of the pair of duplicate spots was analyzed, and the changes of signal intensity in protein levels (5 min exposure time) were plotted (Figure 11). We found that several angiogenesis-related proteins were upregulated by CM, including ANG-2 (Angiopoietin-2), Endoglin, Endostatin, Endothelin-1, Pentraxin 3, PIGF, PAI-1 (Serpin E1), TMP-4, and VEGF. Moreover, the downregulated DPPIV/CD26 showed an expression inhibition in VECs cultured in CM. These results suggest that CM altered the angiogenic balance in the tumor microenvironment by the modulation of proteins’ expression. Interestingly, the increased expression of 23 angiogenesis-promoting proteins (Activin A, Ang-2, Artemin, tissue factor, EG-VEGF, CXCL16, GDNF, GM-CSF, Endothelin-1, Endoglin, IGFBP-1 -2 and -3, PAI-1, PIGF, IL-1β, CCL2, CCL3, MMP-8, MMP-9, NRG1-β1, uPA, and neuregulin-1) from undetectable levels was observed in VECs exposed to MCF-7 CM but not in LECs (Figure 12).

### 2.8. Angiogenesis Proteome Profiler in LECs

A balance between pro- and anti-angiogenic factors are critical for controlling angiogenesis and lymphangiogenesis in response to the tumor. Thus, we examined whether CM regulated the angiogenic factor secretion from MCF-7 cells. Using angiogenic protein profile array, we found that CM strongly decreased the expression of Endostatin/Collagen XVIII (59%), IL-1β/IL-1F2 (45%), Serpin B5/Maspin (62%), PlGF, and VEGF (~80%). On the other hand, the expression of seven angiogenesis-related proteins was increased in LECs by CM, including angiopoietin-2, DPPIV/CD26, Coagulation Factor III, Endoglin/CD105, Endothelin-1, Pentraxin 3, and uPA by 25% ± 10%. TIMP-1, MMP-8, IGFBP-2, and CXCL8 were greatly overexpressed (Figure 12). The protein levels were quantified at two different exposure times (2 min and 4 min) to capture more- and less-expressed proteins. Compared with VECs and consistent with the growth inhibitory actions of CM in LECs, mild modulation in angiogenesis-modulating factors was observed in LECs.

### 2.9. Role of TGF-β and VEGF on MCF-7-CM-Mediated Growth in VECs and LECs

**Figure 13:**
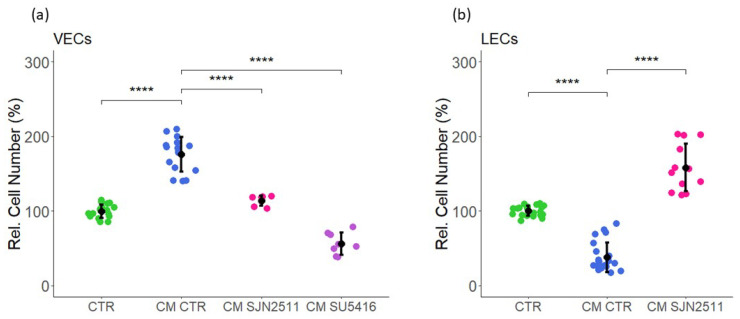
**Effects of VEGF-R antagonist (SU5416) and TGF-β type I receptor antagonist (SJN2511) on CM modulated proliferation of VECs and LECs.** VECs (**a**) and LECs (**b**) were cultured in CM supplemented with the antagonists for 48 h, and cell proliferation was assessed by cell counting. Experiments were performed at least three times in triplicates or quadruplicates, and the data represent mean ± SD. **** *p* < 0.001 compared with the respective control.

The results from the tube formation assay demonstrate that CM induced growth and angiogenesis in VECs. Since VEC growth is induced by TGF-β and VEGF, we investigated whether their presence in MCF-7 CM, in part, contributed to VEC growth. TGF-β, a biphasic growth modulator, is an important factor that increases neo-angiogenesis in tumors and promotes tumor progression [41,42]. To study its potential role in mediating the growth effects of CM on VECs proliferation, we used SJN2511 (a TGF-β type I receptor ALK5 inhibitor). As shown in Figure 13, the CM CTR-induced growth of VECs was abrogated in the presence of the TGF-β1 receptor antagonist. To investigate the possible contribution of CM-derived VEGF on VEC growth, SU5416 (a VEGF-R tyrosine kinase inhibitor) was used in combination with CM, and the effects on the proliferation investigated. Interestingly, the stimulatory effects of CM CTR were reversed from 176 ± 23 to 114 ± 7% by SJN2511 (inhibition of 62%) and from 56 ± 15 by SU5416 (reduction of 120%), respectively (Figure 13a, left panel). VEGF stimulates VECs proliferation and is known to stimulate angiogenesis/neovascularization during the tumor development [43]. Our findings suggest that the presence of VEGF in MCF-7-derived CM is involved in promoting VEC growth. Taken together, our findings suggest that both VEGF and TGF-β in MCF-7 secretome, in part, contribute to CM-induced VEC proliferation.

In contrast to VECs, the growth of LECs was strongly inhibited by MCF-7 CM. Since TGF-β has been shown to inhibit lymphangiogenesis and lymphatic endothelial cell growth [44], we investigated its potential role in mediating the effects of MCF-7 CM on LEC growth. The treatment of LECs with CM CTR inhibited cell proliferation, and these effects were significantly abrogated in the presence of the TGF-β1/ALK5 inhibitor, SJN2511. The effects of CM CTR on LEC proliferation were reversed from 38 ± 20 inhibition to 158 ± 32% induction by the TGF-β1 antagonist, SJN2511 (Figure 13b, right panel). Taken together, we demonstrated that TGF-β plays a critical role in inhibiting LEC growth and may contribute to the observed differential effects of MCF-7 CM in VECs and LECs.

## 3. Discussion

The tumor’s microenvironment plays an active role in promoting breast cancer cell and tumor growth. Secretome from cancer cells, vascular endothelial cells, lymphatic endothelial cells, local fibroblasts, and immune cells contribute to the tumor’s microenvironment. Although the impact of VECs and LECs on breast cancer cell growth has been studied, little is known about the impact of BC cell secretome on VECs and LECs. In our study, using secretome/conditioned medium of breast cancer cells (MCF-7), we provided the first evidence that MCF-7 secretome differentially modulates VEC and LEC growth. The simultaneous treatment of VECs and LECs with MCF-7 secretome induced VEC growth (proliferation, capillary formation, and cell migration) but inhibited LEC growth. The differential growth effects of MCF-7 CM were also evident on the activity/phosphorylation of key signal transduction molecules ERK1/2 and Akt, which were induced in VECs and downregulated LECs. The secretome collected from MCF-7 cells treated with E2 did not further enhance the effects of CM on VEC and LEC growth. A transcryptomic analysis of genes modulated by CM in VECs and LECs revealed a ≈ 10-fold higher number of genes were modulated in LECs (510 genes in VECs versus 5956 genes in LECs). A pathway enrichment analysis of modulated genes revealed significant regulation of pro-growth/angiogenic and cell cycle regulatory pathways in VECs. In contrast to VECs, in LECs, CM downregulated genes for endothelial growth, metabolism (mitochondrial metabolism), and oxidative phosphorylation and upregulated genes that negatively regulate cell cycle progression. Consistent with the changes in gene expression profile, angiogenesis proteome arrays revealed that treatment of VECs with CM upregulated multiple pro-growth and angiogenic proteins, but this was not the case with LECs. Importantly, CM upregulated the expression of 26 proteins that were undetectable in VECs under control conditions. These findings suggest inherent differences in VEC and LEC phenotypes may contribute to the differential effects of MCF-7 secretome on their growth. Interestingly, the TGF-β1 gene was upregulated in VECs, and the growth-promoting and growth-inhibitory actions of CM on VECs and LECs, respectively, were blocked/reversed in the presence of the TGF-β1 receptor antagonist SJN2511. Since TGF-β1 induces VEC growth [45] and inhibits LEC growth [44], it may play a critical role in mediating the differential effects of CM VEC and LEC growth. Finally, the inhibitory effects of CM on the metabolic activity of LECs were also reflected by inhibition of cellular ATP levels, suggesting that CM mediated its growth inhibitory actions, in part, by inhibiting cellular oxidative/mitochondrial metabolism. In conclusion, our findings provide the first evidence that MCF-7 secretome differentially effects VEC and LEC growth. The growth-inducing and growth-inhibitory effects of secretome on VECs and LECs, respectively, are most likely dependent on the intrinsic characteristics of the cells and the generation autocrine/paracrine factors such as TGF-β1 and cellular metabolism. Our findings suggest that the MCF-7 secretome may promote cancer cell/tumor growth by promoting the vascular capillary growth necessary for nutrient supply and by inhibiting lymphatic angiogenesis to limit immune attacks against invading cancer cells.

Despite decades of laboratory and clinical research, BC remains the most common cancer diagnosed in women in the world, including the United States [46,47]; moreover, BC can also occur in men. The growth and metastasis of cancerous mammary epithelial cells are governed by the interaction with vascular angiogenesis and lymphangiogenesis, which are an integral part of the growing tumor, and by the cells/tissues in close proximity, for example, mammary fibroblasts and circulating cells. The tumor’s microenvironment, created by the locally generated paracrine factors, plays a decisive role in tumor progression, and better understanding of its role has led to therapeutic approaches to limiting tumor growth by targeting the cancer cell’s growth directly or indirectly through targeting angiogenesis and lymphangiogenesis, as well as the immune system. With regards to ER-positive BC, which represents the most common type of BC, E2 promotes cancer cell growth by interacting with the estrogen receptor alpha (ERα) and estrogen receptor beta (ERβ) [47]; moreover, drugs such as Tamoxifen, which antagonizes ER, are effective in blocking BC’s progression and increasing patients’ survival [48,49,50]. Since estrogen also influences angiogenesis, its role in the pathophysiology of BC and tumor progression remains a subject of key interest for developing better therapeutic drugs.

Angiogenesis and lymphangiogenesis play a dynamic role in facilitating breast tumor growth and metastasis [7,17]. The role of angiogenesis in supporting tumor growth has been intensively studied and is well-established. However, relatively less is known regarding the role/contribution of lymphangiogenesis/LECs in BC, except for its role in immune cancer cell-counteraction and metastasis. The presence of LECs in tumors has led to the belief that similar to angiogenesis, lymphangiogenesis results from existing lymphatic vessels, which evolved following PROX-1 expression in ECs [7,17]. The tumor’s microenvironment and BC-derived paracrine factors are thought to promote lymphangiogenesis. However, recent findings suggest that tumor LECs may be of different origins. Some tumor-associated LECs express non-venous origins, such as mesenchymal origin [7,17], and marrow-derived progenitors with markers for myeloid LEC progenitors. Interestingly, the presence of TIE-2-expressing monocytes within lymphatic vessels of BC has also been reported [7,17]. Together, these observations also raise the question whether similar to VECs, lymphangiogenesis is driven by BC-derived paracrine factors. Furthermore, whether LECs actively grow into tumors or their intra-tumor presence is simply a result of cancer cells growing around preexisting lymphatic vessels remains unclear. At a functional level, the role of the lymphatic vessel in metastasis is also unclear and under debate. Moreover, unlike vascular capillaries, intra-tumor lymphatic capillaries look unhealthy, with collapsed and disheveled morphology [17]. Interestingly, both angiogenesis and lymphangiogenesis have been shown to be downregulated in primary breast cancer [51]. Compared with the contribution of VEC angiogenesis in tumors, very little is known about the role of LEC/lymphangiogenesis in promoting growth.

Our finding that the MCF-7 secretome induces VEC growth, capillary formation, and cell migration is consistent with its postulated role in supporting tumor growth by supplying nutrients and oxygen. However, our observation that the MCF-7 secretome inhibited LEC growth suggests that MCF-7-derived factors do not contribute to lymphangiogenesis and may facilitate tumor or cancer cell growth via different mechanism(s). It has been shown that LEC-derived factors promote MCF-7 and tumor growth, and these effects are driven by growth factors PCGF-BB and EGF [52]. Although tumor size and new blood vessel formation was obvious, the development of new lymphatic capillaries was not investigated. Since the lymphatic system and the LECs act as a unique immune organ [53], it is feasible that the MCF-7 secretome facilitates cancer cell survival by limiting lymphatic growth and preventing an immune system attack against invading cancer cells. Although provocative, based on our findings and recent reports of LECs’ immune role, this hypothesis needs to be further tested.

The effects of therapeutic agents on cancer cells are not always efficient and points towards the necessity to consider the whole tumor’s microenvironment, in which there are cell-secreted products, growth factors, and proteins [54]. Many studies have investigated the role of vascular and lymphatic ECs in driving breast cancer and tumor growth [52]. However, compared with vascular ECs, the impact of MCF-7-derived paracrine factors in modulating lymphatic EC growth remains unclear. Using conditioned medium from MCF-7 cells, we simultaneously assessed the role of MCF-7-secreted factors on regulating the growth of any vascular lymphatic ECs. Moreover, to dissect the underlying mechanisms involved, we conducted microarray assays and transcriptomic analysis in cells exposed to CM. Furthermore, we assessed the modulatory effects on key signal transduction mechanism(s) as well as changes in the angiogenesis-associated proteins using western blotting and proteome arrays. Since E2 has been associated with BC and induces tumor growth, we also investigated the effects of CM collected from MCF-7 pre-treated with or without E2. Our finding that MCF-7 secretome from E2-treated MCF-7 slightly increased the stimulatory and inhibitory effects of CM on VEC and LEC growth, respectively, suggests that E2 can enhance the generation of paracrine factors responsible for modulating VEC and LEC growth. However, CM itself is potent enough to induce maximal effects, and long-term treatment with E2 may be required to see bigger effects.

Since neovascularization is a critical event for vessel formation and tumor growth, endothelial cell migration constitutes a major step of angiogenesis. The notion that CM acts as a major factor that modulates endothelial migration/proliferation during pathophysiological processes has been widely investigated. Our finding that CM from MCF-7 cells treated with or without E2 induce opposite effects on vascular and lymphatic endothelial cell proliferation suggests that cancer cells may selectively promote angiogenesis but limit lymphangiogenesis. Indeed, the development of new vessels would facilitate nutrient supply to growing cancer cells, whereas the inhibition of lymphatic vessels would prevent the efficient passage of immune cells to counteract the cancer cells. It is well-known that lymphatic and vascular ECs differ both functionally and morphologically, even though they arise from a common embryonic cell source [7,17]. Our finding that MCF-7-derived CM differentially affects LEC and VEC growth suggests that the ECs selectively respond to factors in CM. Indeed, El-Badawy et al. [55] demonstrated that soluble factors in CM could reprogram mesenchymal stromal cells; moreover, similar growth-modulating effects of CM have been observed in various cells associated with breast cancer. These findings suggest that MCF-7-derived soluble factors may play a key role in regulating tumor growth and metastasis by facilitating the dynamic interaction with various cells within the tumor architecture.

Our observation that soluble factors in CM induce VECs proliferation but inhibit LEC growth was unexpected. Since the same CM was used in the two cell types, differences in soluble factors cannot be attributed to these contrasting effects. The E2-treated CM further enhanced the proliferative and anti-proliferative actions in VECs and LECs, respectively. Interestingly, with an in vitro capillary formation assay, we saw a significant increase in capillary formation of VECs treated with CM (CM CTR and CM E2) but not in LECs, suggesting that the difference in response to CM is an inherent characteristic of the cells. It is well-known that LEC and VEC growth is regulated by specific VEGF isotypes [26], suggesting that the generation of soluble factors may have selective effects on lymphangiogenesis and angiogenesis. This contention is also reflected in our finding that MCF-7-derived CM differentially modulated MAPK and Akt phosphorylation in VECs and LECs.

MAPK and Akt are key signal transduction pathways that mediate the growth effects of effector molecules. The MAPK signaling pathway activation regulates several cellular activities, such as proliferation, migration, cell survival, and apoptosis [56] induced by pro-angiogenic factors [57,58]. Like MAPK, Akt is also a key intracellular signaling involved in multiple vital cellular processes [59], and its activation is frequently involved in human malignancy [60]. In our study, we demonstrated that CM differentially modulated ERK1/2 and Akt phosphorylation in VECs and LECs. Consistent with the growth-stimulatory effects of CM in VECs, we observed the stimulatory effects of CM on ERK1/2 and Akt phosphorylation. Moreover, consistent with the growth-inhibitory effects of CM on LEC growth, CM inhibited ERK1/2 and Akt phosphorylation. Since the activations of ERK1/2 and Akt are key drivers for angiogenesis, our findings suggest that MCF-7 CM would promote vascular angiogenesis, but not lymphangiogenesis. Song and Finley [61] showed that VEGF binds to its receptor to initiate MAPK signaling and phosphorylate ERK, and it promotes cell proliferation in the early stages of angiogenesis. ERK signaling also plays a role in disrupting the anti-proliferative effects of ligands such as TGF-β [62]. Whether the inhibitory effects of CM on MAPK and Akt phosphorylation in LECs facilitate cancer cell survival by limiting an immune attack against invading cancer cells needs to be further investigated.

We screened the modulatory impact of MCF-7 secretome on VEC and LEC genes. Our finding that, compared with VECs, CM modulates 10-fold more genes in LECs suggests that the differences in response are likely due to the intrinsic characteristic of LECs and VECs. Moreover, the differences in gene expression may have pathophysiological relevance to cancer/tumor growth. Since CM induced VEC growth and capillary formation, we further analyzed gene expression in VECs in order to better define differentially expressed genes belonging to the “angiogenesis”, “blood vessel formation”, “proliferation”, and “vasculature development” ontology groups during the long-term primary in vitro culture. Our findings highlight the role of key genes belonging to specific functional pathways involved in cell growth and angiogenesis; moreover, they define their functional role in MCF-7-derived CM-treated cells as compared to its relative control. A comparative analysis of the transcriptomes between the two groups revealed valuable information of candidate genes. We also assessed the key genes and pathways involved in the development and progression of vessels/EC growth in breast cancer. A total of 510-DRGs, including 180 downregulated DRGs and 330 upregulated DRGs, were identified in VECs that were exposed to CM. A bioinformatics analysis on DRGs, including GO term enrichment analysis, KEGG pathway analysis, and BioPlanet, found CM-associated genes and pathways, which exerted effect on cancer growth and progression from different sides. The majority of the DRGs were functionally enriched in the cell cycle, integrated BC pathway, polo-like kinase 1 (PLK1) pathway, p73 transcription factor network, Aurora B signaling, lysosome, kinesins, and FOXM1 transcription factor network.

The high impact of CM on VECs is apparent from the most significant enriched pathway: the Polo-like kinase 1 (PLK1) pathway (adjusted *p*-value 1, 9.9 × 10^−4^). PLK1 plays an important role in the initiation, maintenance, and completion of mitosis [63], and both the up- or downregulation of PLK1 can induce defects in mitosis and cause tumorigenesis [64,65,66]. The mammalian transcription factor Forkhead Box M1 (FoxM1) plays an important role in regulating mitotic entry and subsequent execution of the mitotic program by controlling the expression of a cluster of G2/M target genes. PLK1 binds directly to FoxM1, resulting in the enhanced expression of key mitotic regulators [67,68,69]. The genes involved in polo-like kinase 1 (PLK1) pathway and FOXM1 transcription factor network are mostly downregulated, including PLK1, CDC20, AURKA, CCNA2, CCNB2, CDK1, TPX2, PRC1, KIF20A, ECT2, BUB1, and NDC80. The listed genes exhibit oncogenic functions, and thus the inhibition of these genes’ expression may contribute to the tumor treatment and provide some basis for the development of new targeted drugs [70,71,72,73,74,75,76,77,78,79].

Regarding LECs, 5956 DRGs were identified in three datasets, and a BioPlanet process analysis showed that the DEGs were mainly enriched in the processes of the mitotic phases’ transition, including programmed cell death and apoptosis, which are important to block cancer cell proliferation [80]. Moreover, the enrichment of genes involved in protein metabolic processes and ribosome biogenesis that play crucial role in the development and progression of spontaneous cancers [81] and in the control of cell growth and proliferation [82] were evident. Because DNA replication is central to cell proliferation, its dysregulation can cause cancer, mitochondrial damage, and apoptosis. Thus, it is a fundamental biological process [83,84,85], as well as rRNA [86] and ncRNA [87], tRNA [88], and mRNA processing [89], in which alteration in mRNA can contribute to tumor progression by the expression of oncogenes or tumor-suppressor genes. These results indicated that the biological characteristics of LECs cultured in CM were mainly associated with negative regulators of cell cycle or pathways that regulate cell proliferation.

Recent studies provide evidence that endothelial metabolism and mitochondrial respiration play an essential role in regulating EC growth and angiogenesis [30,31,32]. Since many of the top 20 genes modulated by CM in LECs were linked to metabolism, we further assessed the changes in genes associated with glycolysis, one C metabolism/nucleotide synthesis, Urea/TCA cycle, and oxidative phosphorylation. In CM-treated LECs, many genes associated with metabolism, mitochondrial respiration, and oxidative phosphorylation were downregulated, suggesting that CM limits LEC growth by inhibiting LEC metabolic activity. In contrast to LECs, CM did not inhibit metabolic activity in VECs, suggesting that differences in LEC and VEC metabolism in response to CM may, in part, be responsible for the differential growth. The inhibitory effects of CM on LEC metabolism were also reflected by a significant decrease in ATP synthase gene expression and ATP levels in CM-treated LECs. Interestingly, it has recently been shown that mitochondrial respiration controls the Prox1-VEGFR3 feedback loop during LEC fate-specification and maintenance [32]. The observations herein provide the first evidence that breast cancer secretomes differentially modulate metabolism in LECs and VECs. Since mitochondrial respiration/metabolism regulates hypoxia and vice versa [90], it is tempting to speculate that CM may create a hypoxic response in LECs which may activate angiogenesis in neighboring VECs.

Consistent with our findings that MCF-7 CM induces VEC growth, capillary formation, cell migration, and upregulates genes and pathways that promote angiogenesis, we also observed upregulation of several pro-growth/pro-angiogenic proteins in protein arrays. In VECs, the treatment of MCF-7 CM upregulated the expression of 26 proteins from undetectable levels. The majority of upregulated proteins were those with known proangiogenic effects, such as activin A, angiopoietin-2, artemin, EG-VEGF, HB-EGF, GM-CSF, CXCL16, GDNF, MMP-8 and -9, neuregulin-1, placental growth factor (PIGF), endothelin-1, plasmin activator, TF, IGFBP-1, IGFBP-2, IGFBP-3, CCL-2, CCL3, and IL-1β. However, some proteins which negatively regulate angiogenesis (TIMP-1, TIMP-4, TSP-1, vasohibin, Serpin 1, endostatin, and pentraxin 3) were also upregulated, suggesting that the balance between the pro- and anti-angiogenic proteins may define the overall pro-angiogenic effects of CM in VECs. Interestingly, in contrast to VECs, very few angiogenesis-associated proteins were regulated by CM in LECs. The pro-angiogenic proteins that were noticeably downregulated by CM in LECs were 1L-1β, IGFBP-1, FGF-2, VEGF, and PIGF. Moreover, an increase in antiangiogenic proteins pentraxin-3 and TIMP-1 was also observed. Together, these findings suggest that in LECs, MCF-7 CM shifts the balance toward anti-angiogenic molecules, whereas in VECs, the balance is shifted toward pro-angiogenic molecules.

Among the top 10 genes upregulated by CM in VECs, TBF-β1 was the most prominent. Since TGF-β1 has been shown to induce vascular EC growth [45,91] and inhibit lymphangiogenesis and LEC growth [44], we investigated its potential role in mediating the differential growth effects of CM in VECs and LECs. Our finding that the growth-stimulatory effects of CM in VECs and growth-inhibitory effects in LECs were abrogated/reversed in the presence of the TGF-β1 receptor antagonist SJN5411 suggests that it may play a key role in defining the differential growth effects of MCF-7 CM in VECs and LECs. Since TGF-β1 mediates its effects in part via VEGF [45], we investigated whether it is also involved in mediating the pro-angiogenic effects in VECs. The treatment of VECs with CM in the presence of VEGF-R kinase inhibitor SU5416 blocked the stimulatory effects of CM on VEC growth. Taken together, these findings provide evidence for a distinct role of TGF-β1 in mediating the differential growth effects of CM in VECs and LECs. Since CM also differentially influenced genes for mitochondrial metabolism, we speculate that CM-driven changes in metabolic activity may also be TGF-β1-mediated and need to be further investigated. The fact that TGF-β1 reversed the growth effects of CM in VECs and LECs reaffirms that our findings were not an artifact or culture conditions.

The presence of Prox-1 is a key characteristic of LECs and separates them from VECs. Prox-1 plays an important role in driving lymphangiogenesis and LEC growth [7,17]. Hence, it is feasible that the inhibitory effects of MCF-7 secretome on LEC growth are mediated via factors and genes known to regulate Prox-1. In this context, our observation that the TGF-β1 antagonist reverses the inhibitory effects of CM is supported by the fact that TGF-β1 inhibits lymphangiogenesis and downregulates Prox-1; moreover, the TGF-β1 antagonist reverses these effects [44]. Interestingly, mitochondrial respiration is a key regulator of Prox-1 [32], and TGF-β1 attenuates mitochondrial bioenergetics in ECs [32]. Moreover, the tumor-derived TGF-β was shown to inhibit mitochondrial respiration and suppress IFN-γ by CD4+ cells [92], suggesting that TGF-β1 may play a key role in mediating the inhibitory effects of CM on LECs. Indeed, consistent with the above facts, we observed the significant downregulation of metabolism, including mitochondrial respiration, in CM-treated LECs but not in VECs. Moreover, in the top 10 genes that were upregulated was ANKRD1, which is a target gene for YAP and TAZ and is known to negatively regulate Prox-1 [93]. Importantly, YAP/TAZ hyper-activation represses Prox-1 and negatively affects lymphangiogenesis [93]. Additionally, IFI6, which was upregulated by CM in LECs, has been shown to regulate mitochondrial mechanism [94]. Other key genes that were regulated by CM in LECs and modulate endothelial metabolism were COX7A2, COX7B, COX7C, CPX6A1, COX8A, COX5A, COX5B, MTHFD2, PGK1, PKM, NDUFB7, NDUFA7, ATP5D, ATP5G1, ATP5A1, ATP5J2, SRM, and SDHD [30,31]. In addition to the genes linked to Prox-1 and LEC metabolism, the top ten downregulated genes in LECs that are known induce angiogenesis were metallothionein -1A, -1B, -1X, -1L [95], ID-1 [96], and IFI6 [94]. The top 10 EC metabolism-associated genes upregulated by CM in LECs were ASNS, CBS, GYPC, MTHFD2, PSAT1, and STC2 [30,33,34], suggesting that CM may influence LEC growth and lymphangiogenesis by regulating metabolism associated with Prox-1. Interestingly, we observed that MCF-7 secretome upregulated MTHFD2, a folate-cycle enzyme shown to induce cancer immune evasion [97]. Since the lymphatic system also has an immunomodulatory role to counteract cancer cells, combined with the fact that metabolism influences immune action, it is tempting to speculate that MCF-7 secretome upregulates MTHFD2 in LECs to evade and survive the immune insult.

Recent studies have shown that LECs’ secretome induces the growth of breast cancer cells and tumors [90]. The pro-growth effects observed were mediated via increased release of growth factors (PDGF BB and EGF) from CM-treated or CM-educated LECs. In our study, we did not investigate the effects of LEC-derived CM on cancer cell growth. Moreover, the experimental setup and treatment conditions were different. In our studies, we assessed the effects of MCF-CM in presence of 0.4% serum, whereas Lee et al. [52] assessed the effects of TCM in presence of 30% TCM and 70% endothelial growth medium. Hence, the differences in culture conditions may explain the differences in growth factors generated by LECs. In our study, we used CM from MCF-7 cells. However, whether these effects are also mimicked by the CM of other BC cells remains unknown and should be further investigated. Findings from Lee’s lab [98] showed that the effects of CM from different BC cells, including MCF-7, on LEC marker hCCL5 vary considerably. Hence, the observed effects of MCF-7 CM on LEC and VECs may or may not be mimicked by other BC cells and should be further investigated.

Cancer cells alone are not able to completely reproduce the original tumor, and tumorigenesis process involves interplay with VECs that form capillaries to supply oxygen and nutrients to the tumor, as well as LECs, which create capillaries to allow immune cell passage early on as well as metastasize at a later stage. Hence, lymphangiogenesis and angiogenesis are crucial phenomena involved in the spread of cancer cells, and new discoveries and advancing knowledge on these phenomena will allow an improvement in the treatment of cancer patients. The angiogenic growth of lymphatic vessels drives several biological processes that allow cancer cells to access systemic circulation to metastasize. To estimate the more general effects of angiogenic proteins, we conducted proteome profiling assays of LECs cultured in conditioned medium. Among the angiogenesis-related proteins, noticeable downregulation of pro-angiogenic molecules (IL-1β, IGFBP-1, and FGF-2) and upregulation of anti-angiogenic molecules (Pentraxin 3 and TIMP-1) were observed. However, the marginal increase in some pro-growth and decrease in some anti-growth molecules (Serpin B5, MMP-8, CXCL-8, endostatin, and Ang-2) was also observed. Since the magnitude of the CM effects on LEC metabolism was much higher, it is feasible that CM inhibits LEC growth by influencing LEC metabolism.

Among the key molecules regulated by CM were angiopoietin-2 (Ang-2), which is involved in the development of lymphatic vessels [99]. It enhances angiogenic and anti-apoptotic activities of LECs via the Tie2/Akt signaling pathway [100] and participates in the control of lymphatic metastasis. Interestingly, PTX3 has been shown to inhibit the growth and vascularization of FGF-dependent tumors and cell proliferation [101], whereas TIMP-1 represses lymphangiogenesis [102]. The overexpression of DPPIV was associated with a decrease of migration and invasion in several malignancies [103,104]. Therefore, these proteins could act as tumor suppressors. As a marker for tumor diagnosis, prognosis, and therapy, Endoglin/CD105 is involved in vascular development and remodeling by enhancing cell proliferation and migration [105,106]. However, very little is known about its expression and its role in lymphatic vessels. Matrix metalloproteinases (MMPs) play an important role in cancer progression and metastasis, which makes them an attractive target for cancer therapy. In our proteome profiler assay, MMP8 was the most overexpressed protein (156%) and is reported to have onco-suppressive properties in several tumors, reducing cell proliferation and invasion [107,108].

A proteome profile assay determined the presence of five proteins were strongly downregulated: (1) Endostatin/Collagen XVIII and (2) IL-1β/IL-1F2, which have anti-metastatic, anti-angiogenic potency [109,110,111], and blocking their function may suppress tumor progression and lymphangiogenesis; (3) PlGF that stimulates the growth and migration of endothelial cells, contributing to both neovascularization and lymphangiogenesis [112,113]; (4) Serpin B5/Maspin, correlated with tumor development and aggressiveness [114], which possesses tumor-suppressing activity against breast tumor growth and metastasis [115]; (5) VEGF, an inducer of angiogenesis and lymphangiogenesis, and the first VEGF inhibitor has recently entered the clinic for treatment of cancer [116,117]. The fact that CM inhibited LEC growth even though it downregulates many growth-inhibitory proteins such as endostatin suggests the involvement of multiple mechanisms and factors in driving CM-mediated LEC’s growth inhibition. An overall balance between pro- and anti-growth factors/cytokines and mechanisms may play a critical role in defining the final effects of MCF-7 secretome on LEC growth.

In summary, our results provide the first evidence that MCF-7 secretome differentially modulates growth of VECs and LECs. Our findings implicate the involvement of different mechanisms in mediating the pro- and anti-growth effects of CM in VECs and LECs. The mitogenic effects of CM on VECs involves multiple well-established EC growth factors and cytokines, whereas the antimitogenic actions of CM in LECs involves cellular/mitochondrial metabolism linked to Prox-1. The differential effects of CM in VECs and LECs seem to involve TGF-β1 and involve MAPK as well as Akt. Our findings provide evidence that paracrine factors in MCF-7 CM can actively regulate VEC and LEC growth. Moreover, a transcriptomic analysis of microarray data in our study provides a comprehensive bioinformatics gene list, as well as an analysis of DRGs that may be involved in the development and progression of BC. These findings may act as potential biomarkers and novel therapeutic targets. However, further studies are required to confirm the function of the identified genes and pathways in vitro and in vivo. Furthermore, the DRGs identified in our study could serve as candidates for potential molecular targets for the diagnosis and treatment of BC. Finally, soluble factors/proteins in CM, such as TGFβ1, can be simultaneously targeted to block neovascularization and provide optimal treatment against breast cancer. Our observations may provide valuable information to further investigate the role of BC secretome in breast cancer/tumor development. The identified genes and proteins could serve as attractive therapeutic targets for counteracting vascular angiogenesis and modulating the lymphatics in tumors.

## 4. Materials and Methods

### 4.1. Cell Culture

Human umbilical vein endothelial cells (VECs) were purchased from Lonza (Walkersville, MD, USA). The VECs were cultured in collagen (rat tail, 5 μg/cm^2^) 75 cm^2^ flasks under standard tissue culture conditions (37 °C, 5% CO_2_) in complete growing media: EBM-2 (Endothelial Basal Medium-2) supplemented with Glutamax (1×), antibiotic-antimycotic (AA: 100 μg/mL streptomycin, 100 μg/mL penicillin, and 0.025 μg/mL amphotericin B), LSGS (2% *v/v* FCS, 1 µg/mL hydrocortisone, 10 ng/mL human epidermal growth factor, 3 ng/mL human basic fibroblast growth factor, and 10 µg/mL heparin), and 10% FCS (fetal calf serum). The medium was changed every two days until sub-confluency. Cells were between the 4th and 12th passages were used for the experiments.

Human dermal microvascular endothelial cells, neonatal and pooled (LECs; >90%), were acquired from Lonza (Walkersville, MD, USA). The LECs were propagated in EGM™-2MV BulletKit™ Growth Medium (Walkersville, MD, USA) containing EBM-2 (Endothelial Basal Medium-2) supplemented with human epidermal growth factor (hEGF), vascular endothelial growth factor (VEGF), R3 insulin-like growth factor-1 (R3-IGF-1), ascorbic acid, hydrocortisone, human fibroblast growth factor-Beta (hFGF-β), fetal bovine serum (FBS), and Gentamicin/Amphotericin-B (GA). The medium was changed every two days, and the Reagent Pack (CC5034, Walkersville, MD, USA), containing Trypsin/EDTA, Trypsin Neutralizing Solution, HEPES Buffered Saline Solution, was used for passaging (between the 4th and 8th passages).

The MCF-7 human breast cancer cell line (mammary epithelial cells) was provided by Dr André Fedier (Clinic for Gynecology, University Hospital, Zurich) and was obtained from the ATCC (American Type Culture Collection). The MCF-7 cells were cultured in standard tissue culture conditions (37 °C, 5% CO_2_) in DMEM/F12 medium supplemented with Glutamax (1×), antibiotic-antimycotic (AA 100 μg/mL streptomycin, 100 μg/mL penicillin, and 0.025 μg/mL amphotericin B), and 10% FCS. The cells were cultured between the 33rd and 48th passages, and the medium was changed every two or three days until sub-confluency.

### 4.2. Conditioned Medium (CM)

**Figure 14:**
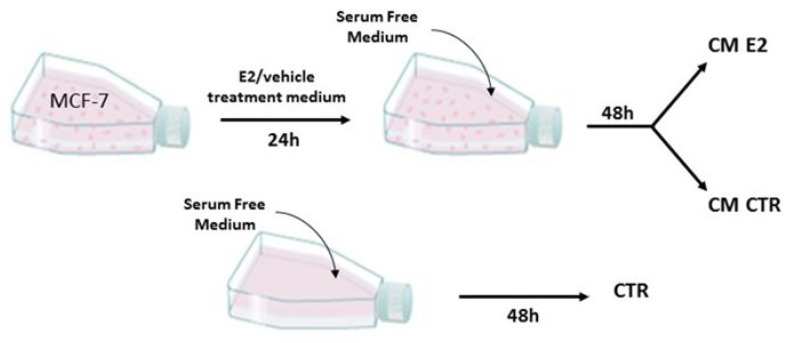
**Scheme outlining control and MCF-7 conditioned medium preparation.** MCF-7 cells were grown on 75 cm^2^ tissue culture flasks and treated with and without E2. The medium was replaced with serum-free medium and collected after 48 h. A serum-free medium added to culture flask without cells, was used as control.

When the MCF-7 cells reached 70% confluence in 75 cm^2^ tissue culture flasks, the cells were washed with HBSS (with Ca^2+^ and Mg^2+^), and the normal growth medium was replaced with E2 treatment medium (DMEM-F12, Glutamax, antibiotic-antimycotic solution, 2.5% steroid free FCS (charcoal-stripped), and E2/DMSO (10 nM)). After 24 h, the cells were washed with HBSS (with Ca^2+^ and Mg^2+^), and the treatment medium was replaced with a serum-free medium (EBM-2, Glutamax, antibiotic-antimycotic solution) for 48 h. The supernatant containing all factors secreted by the cells was harvested from the cultures, centrifuged (5 min, 1000× *g*, room temperature (RT)), and filtered through 0.2 µm syringe filters. The resulting conditioned medium (CM) was aliquoted and either stored at −80 °C or directly used. As a control, we used a serum-free medium added to an empty flask and treated under the same conditions (Figure 14).

### 4.3. Cell Proliferation Assay

An endothelial cell proliferation assay was performed by counting cell numbers. Cells were seeded in a 24-well plate at a density of 2.5 × 10^4^ cells/well and allowed to attach. Next, the cell growth was arrested in a starving medium (EBM-2, Glutamax, antibiotic-antimycotic solution and 0.4% steroid free FCS) for 6 h and then was replaced with CM (supplemented with 0.4% FCS). After 48 h, the cells were trypsinized, transferred in cuvettes, and counted using a Coulter Counter (Coulter Electronics, Luton, UK). All samples were performed in triplicates. The relative cell number was assessed by normalizing to the control.

### 4.4. Microvessel Formation Assay

An angiogenesis μ-slide was coated with ice-cold Matrigel solution and incubated at 37 °C for at least 30 min to allow the Matrigel to solidify. The trypsinized VECs were counted and suspended in 1 mL per each CM at a density of 80,000 cells/mL. They were supplemented with 0.4% FCS; the cells were incubated 30 min prior to the transfer of 50 µL (4000 cells) on top of the gel. The μ-slide was incubated at 37 °C to allow cells to form micro-vessels overnight. Five images of the tubular structures were taken for each well at 10× magnification using an Olympus inverted microscope. The quantification was done measuring micro-vessels’ length using the Olympus Xcellence Pro software (Shinjuku, Japan).

### 4.5. Migration Studies

The cell migration was determined by a scratch/wound closure assay. The VECs were plated in a collagen-coated 24-well plate (Biocoat Collagen I Cellware, Corning, 354408, BioCoat, Kennebunk, ME 04043, USA) and grown to confluence before a scratch was made using a yellow pipette tip. The cells were then washed with HBSS (with Mg^2+^ and Ca^2+^) to remove the cell debris and cultured in CM with 0.4% FCS. Several images of the scratch were taken using an Olympus inverted microscope right after the CM addition (T0) and after 24 h of incubation (T24). The wound closure area was determined using Image J software and calculated accordingly (area T0—area T24)/area T0.

### 4.6. Western Blotting

The VECs and LECs were seeded in 35 mm tissue culture dishes (6 × 10^5^ cells/dish), and after allowing them to adhere, the cells were starved for 7 h. The cells were cultured in CM for 45 min and after washing with cold PBS, they were lysed with a cell lysis buffer (containing 20 mM Tris pH7.5, 1% Triton X-100, 150 mM NaCl, 1 mM EGTA, 1 mM EDTA, 2.5 mM sodium phosphate, 1 mM β-glycerophosphate, 1 mM sodium vanadate, 0.5 PMSF, and 0.2% SDS) on ice, followed by scraping. The samples were homogenized for 2–4 s by sonication and stored at −20 °C until further use. The protein concentration was obtained by absorbance measurement using the Pierce BCA Assay Kit (Thermo Fisher, Waltham, MA, USA) using Tecan Infinite M200 Series Microplate Reader. The cell extracts were separated by 10% SDS-polyacrylamide gels electrophoresis, transferred to a nitrocellulose membrane by wet electro-blotting, and to prevent unspecific binding, the membrane was blocked in 5% milk at RT for 1 h. Next, the membrane was incubated with the primary antibody overnight at 4 °C, washed with 1% milk, and incubated with the secondary antibody at RT for 1 h. The Odyssey LI-COR system (LI-COR, Lincoln, NE, USA) was used to detect protein with IRDyes. The X-OMAT LS films using ECL was used to detect peroxidase-conjugated secondary antibodies on a membrane previously covered with SuperSignal West Dura LuminolSubstrate, which were developed with the CAWOMAT 2000 IR film developer (WIROMA AG, Niederscherli, Switzerland).

For the successive detection of additional protein, the membrane was washed with PBS after analysis of the first protein, incubated for 15 min with the stripping buffer 1 (0.2 M glycine, 0.1% SDS, 1% Tween 20, PH 2.2, in distilled water), and subsequently shortly washed in the stripping buffer 2 (1 M NaCl in PBS). Afterward, the membrane was washed with PBS for 30 min, and the protein detection procedure restarted from the blocking process.

### 4.7. Microarray

For the microarray analysis, the endothelial cells were grown and starved as described in the western blot section above. The starving media was replaced with CM and supplemented with 0.4% FCS. After 20 h of CM treatment, the cells were lysed using RNA Lysis buffer (Zymo Research, Irvine, CA, USA) and frozen at −80 °C until further use. The total RNA was extracted using the Quick-RNA MiniPrep Kit (ZymoResearch, Irvine, CA, USA, R1055) according to the manufacturer’s instructions. The RNA quality and quantity were assessed by the absorbance at 260 nm using Tecan Spectrofluorometer reader (Infinite 200 NanoQuant). The samples were frozen at −80 °C, and a microarray analysis was performed using Affymetrix Clariom S Assay, human (Applied Biosystems by Thermo Fisher Scientific Inc., Waltham, MA, USA, 902927). For the transcriptome analysis, fragmented biotin-labeled ds cDNA was hybridized to Clariom™ S arrays (Clariom™ S arrays, human). For scanning, the Affymetrix Gene-Chip Scanner-3000-7G was used, and the image and quality control assessments were performed using GeneChip Command Console Software (GCC) v5.0. The transcriptome analysis was conducted at the transcriptomic core facility at the Center for Molecular Medicine Cologne (CMMC). For the gene expression analysis, the CEL files obtained from the microarray experiments were uploaded in the Transcriptome Analysis Console (TAC, Applied Biosystems by Thermo Fisher Scientific Inc, OK, USA). Genes with a fold change ± 1.5 and FDR *p*-value < 0.05 were selected for further analysis. The pathway analysis was performed using the Enrichr website [118,119]. The microarray data were deposited in the public Gene Expression Omnibus (GEO) database under the accession no. GSE189084 (https://www.ncbi.nlm.nih.gov/geo/query/acc.cgi?acc=GSE189084) (accessed on 25 May 2022).

### 4.8. Quantitative RT-PCR

For the microarray results validation, a quantitative real-time polymerase chain reaction (qRT-PCR) was performed. The samples were cultured in a CM, and RNA isolation was performed as described in Section 2.7 above. The RNA concentration was determined by measuring the absorbance at 260 nm, and the RNA purity was considered by the ratio A260/A280 > 1.8 and A260/A230 > 1.8. We used 0.5 μg of total RNA for reverse transcription of each sample using the RT^2^ First Strand Kit (Qiagen) according to the manufacturer’s instructions. Gene expression and detection were performed on a Bio-Rad CFX96 Real-Time PCR Detection System using Custom RT^2^ Profiler PCR Arrays in a 96-well-plate format according to the manufacturer’s protocol. The PCR reaction was run with 95 °C for 10 min, followed by 40 cycles of 95 °C for 15 s and 60 °C for 1 min. As the internal controls, GAPDH and LDHA were used. The experiment was performed once in triplicates, and the relative gene expression was calculated using the 2^−ΔΔCt^ method.

### 4.9. Angiogenesis Proteome Array

For the analysis of the angiogenesis-related proteins in VECs and LECs cultured in CM, the Proteome Profiler Human Angiogenesis Array Kit (R&D Systems, Minneapolis, MN, USA, ARY007) was used. The cells were seeded in 60 mm tissue culture dishes (~5 × 10^6^ cells/dish), starved, and cultured in CM (0.4% FCS) for 48 h. After trypsinization and centrifugation, the pellet was lysed in lysis buffer 17 (R&D Systems, Minneapolis, MN, USA) and supplemented with glycerol (10%) aprotinin, leupeptin, and pepstatin (10 µg/mL each); and after rocking at 4 °C for 30 min, the lysates were centrifuged at 14,000× *g* for 5 min. The supernatant was collected into clean tubes and frozen at −80 °C until further processing. The ready-to-use membranes were incubated o/n with equal amounts of samples (185 µg in 1.5 mL). The detection of the following proteins was performed according to the manufacturer’s instructions. For the exposure of the membranes, Hyperfilm ECL (Amersham, Zurich, Switzerland) was used in a CAWOMAT 2000 IR film developer (Wiroma AG, Niederscherli, Switzerland). The average signal of the pair of duplicate spots was determined using ImageJ software after background subtraction.

### 4.10. Intracellular ATP Content

The cells were seeded at a density of 5000 cells per well in a 96-well plate and incubated at 37 °C in a humidified atmosphere with 5% CO_2_. After 24 h, the medium was aspirated, and the cells were treated with CM in presence of 0.4% FCS and the respective CTR. After 48 h of incubation for each treatment, half of the replicates were lysed with 75 μL of CellTiter-GLO reagent (Promega), and the ATP content was determined by measuring the luminescent signal. The remainder was lysed in 75 μL of 1% Triton-X 100 in deionized H_2_O (*w*/*v*) for protein determination by bicinchoninic acid (BCA) assay (Interchim, Montluçon Cedex, France). All measurements were performed using the GloMax Discover Microplate Reader (Promega). The luminescent signal was normalized by the protein content and expressed as percentage of the untreated cells.

### 4.11. Statistical Analysis

The data in the present study were expressed as mean ± SD of at least three independent experiments from different cell passage numbers. After the normal distribution was proven via a Shapiro–Wilk test, a parametric test was performed with ANOVA analysis, followed by Tukey’s HSD multiple pairwise comparisons. If the normality test was not passed, a non-parametric test was performed with a Kruskal–Wallis rank sum test and a subsequent pairwise Wilcoxon test with Benjamini–Hochberg corrections. All statistical calculations were run using R-studio, and the values were considered significantly different when *p* < 0.05.

## 5. Conclusions

Our findings suggest that MCF-7/breast cancer cell secretome may facilitate tumor growth by promoting vascular angiogenesis to facilitate nutrient supply and by inhibiting LEC growth, to limit attacks by lymph-derived anti-cancer immune molecules.

## Figures and Tables

**Table 1:** Top ten upregulated genes in VECs cultured in CM.

Gene Symbol	Description	Log2 FC	FDR *p*-Value
TGFBI	transforming growth factor, beta-induced, 68 kDa	2.05	0.0073
ABCA1	ATP binding cassette, subfamily A, member 1	1.85	0.0015
EFNA1	ephrin-A1	1.82	0.0251
TMEM63B	transmembrane protein 63B	1.78	0.0001
CCL20	chemokine (C-C motif) ligand 20	1.73	0.0072
WDTC1	WD and tetratricopeptide repeats 1	1.73	0.0308
EDEM1	ER degradation enhancer, mannosidase alpha-like 1	1.69	0.009
NISCH	nischarin	1.68	0.0008
SORT1	sortilin 1	1.66	0.0007
MGP	matrix Gla protein	1.65	0.0004

Transcriptome Analysis Console (TAC, Applied Biosystems) was used for analyzing gene expression data of VECs cultured in CM. In the table are listed the top ten upregulated genes with the respective fold changes (FC) and adjusted *p*-values (FDR *p*-value). For the analysis, a fold change (FC) cut-off of ±1.5 and an FDR *p*-value of 0.05 were applied.

**Table 2:** Top ten downregulated genes in VECs cultured in CM.

Gene Symbol	Description	Log2 FC	FDR *p*-Value
IL1RL1	interleukin 1 receptor-like 1	−2.64	0.0096
MKI67	marker of proliferation Ki-67	−2.36	0.0001
HIST1H1B	histone cluster 1, H1b	−2.05	0.0001
RRM2	ribonucleotide reductase M2	−1.95	0.0014
ARHGAP11B; ARHGAP11A	Rho GTPase activating protein 11B Rho GTPase activating protein 11A	−1.93	0.0013
KIF20B	kinesin family member 20B	−1.93	0.0045
ANLN	anillin actin binding protein	−1.91	0.0048
TOP2A	topoisomerase (DNA) II alpha	−1.8	0.0001
SHCBP1	SHC SH2-domain binding protein 1	−1.79	0.0117
NCAPG	non-SMC condensin I complex subunit G	−1.77	0.0007

Transcriptome Analysis Console (TAC, Applied Biosystems) was used for analyzing the gene expression data of VECs cultured in CM. In the table are listed the top ten downregulated genes with the respective fold changes (FC) and adjusted *p*-values (FDR *p*-value). For the analysis, a fold change (FC) cut-off of ±1.5 and FDR *p*-value of 0.05 were applied.

**Table 3:** Top ten upregulated genes in LECs cultured in CM.

Gene Symbol	Description	Log2 FC	FDR *p*-Value
ASNS	asparagine synthetase (glutamine-hydrolyzing)	6.03	4.11 × 10^−12^
CBS	cystathionine-beta-synthase	5.02	2.28 × 10^−10^
GYPC	glycophorin C (Gerbich blood group)	5	1.53 × 10^−9^
PSAT1	phosphoserine aminotransferase 1	4.9	7.12 × 10^−11^
MTHFD2	methylenetetrahydrofolate dehydrogenase (NADP+ dependent) 2, methenyltetrahydrofolate cyclohydrolase	4.45	1.29 × 10^−9^
STC2	stanniocalcin 2	3.73	1.27 × 10^−8^
ANKRD1	ankyrin repeat domain 1 (cardiac muscle)	3.51	1.53 × 10^−9^
HOOK3	hook microtubule-tethering protein 3	3.4	3.45 × 10^−9^
ANKRD36B	ankyrin repeat domain 36B	3.29	1.30 × 10^−8^
CHD1	chromodomain helicase DNA binding protein 1	3.26	1.35 × 10^−8^

Transcriptome Analysis Console (TAC, Applied Biosystems) was used for analyzing the gene expression data of LECs cultured in CM. In the table are listed the top ten upregulated genes with the respective fold changes (FC) and adjusted *p*-values (FDR *p*-value). For the analysis, a fold change (FC) cut-off of ±1.5 and FDR *p*-value of 0.05 were applied.

**Table 4:** Top ten downregulated genes in LECs cultured in CM.

Gene Symbol	Description	Log2 FC	FDR *p*-Value
COX8A	cytochrome c oxidase subunit VIIIA (ubiquitous)	−5.88	1.82 × 10^−10^
MT1B	metallothionein 1B	−5.32	1.53 × 10^−12^
MT1A	metallothionein 1A	−5.31	4.11 × 10^−12^
MT1X	metallothionein 1X	−5.27	2.29 × 10^−11^
SNRPD3	small nuclear ribonucleoprotein D3 polypeptide	−5.21	1.53 × 10^−12^
ID1	inhibitor of DNA binding 1, HLH protein	−5.13	2.33 × 10^−8^
MT1L	metallothionein 1L (gene/pseudogene)	−5.1	4.66 × 10^−10^
COX7B	cytochrome c oxidase subunit VII B	−5.03	2.98 × 10^−9^
IFI6	interferon, alpha-inducible protein 6	−4.93	4.91 × 10^−8^
TMEM88	transmembrane protein 88	−4.68	5.32 × 10^−12^

Transcriptome Analysis Console (TAC, Applied Biosystems) was used for analyzing the gene expression data of LECs cultured in CM. In the table are listed the top ten downregulated genes with the respective fold changes (FC) and adjusted *p*-values (FDR *p*-value). For the analysis, a fold change (FC) cut-off of ±1.5 and FDR *p*-value of 0.05 was applied.

**Table 5:** Pathway enrichment analysis (Enrichr) of DRGs between VECs cultured in CM and the relative CTR.

Pathway	Overlap	Adj. *p*-Value
**BioPlanet**		
Polo-like kinase 1 (PLK1) pathway	10/46	1.99 × 10^−4^
Cell cycle	30/453	0.001296906
p73 transcription factor network	10/79	0.010895079
Aurora B signaling	7/39	0.012713335
Lysosome	12/121	0.013134648
SRF and microRNAs in smooth muscle differentiation and proliferation	4/12	0.024709241
M phase pathway	10/96	0.024709241
p53 activity regulation	11/118	0.027319292
Systemic lupus erythematosus	12/139	0.027319292
Phosphorylation of Emi1	3/6	0.029461214
MicroRNA regulation of DNA damage response	8/70	0.036366012
Cyclin A/B1-associated events during G2/M transition	4/15	0.036438016
Kinesins	5/27	0.036987861
FOXM1 transcription factor network	6/41	0.036987861
Integrated breast cancer pathway	12/152	0.036987861
Mitotic prometaphase	6/43	0.044760715
**GO Biological Process**		
mitotic spindle organization (GO:0007052)	17/157	0.001553571
microtubule cytoskeleton organization involved in mitosis (GO:1902850)	15/128	0.001553571
mitotic cytokinesis (GO:0000281)	8/49	0.030235267
**KEGG**		
Lysosome	12/128	1.25 × 10^−4^

Pathway enrichment analysis of DRGs between VECs cultured in CM and CTR. Analysis was performed comparing the BioPlanet, GO Biological Process, and KEGG on the Enrichr website by uploading DRGs obtained by Transcriptome Analysis Console (TAC). In the table are listed the pathways with adjusted *p*-value < 0.05 (*p*-value adjusted for multiple testing).

**Table 6:** List of genes involved in the enriched pathways.

Gene Symbol	Description	Log2 FC	FDR *p*-Value
ANLN	anillin actin binding protein	−1.91	0.0048
AP3B2	adaptor-related protein complex 3, beta 2 subunit	−0.92	0.0372
ATP6AP1	ATPase, H+ transporting, lysosomal accessory protein 1	0.67	0.0482
AURKA	aurora kinase A	−0.72	0.0321
BUB1	BUB1 mitotic checkpoint serine/threonine kinase	−1.53	0.0242
CD68	CD68 molecule	1.29	0.0315
CDC20	cell division cycle 20	−0.99	0.0298
CEP55	centrosomal protein 55kDa	−1.24	0.0149
CLASP1	cytoplasmic linker associated protein 1	1.01	0.0092
CTSA	cathepsin A	0.84	0.03
DLGAP5	discs, large (Drosophila) homolog-associated protein 5	−1.38	0.007
ECT2	epithelial cell transforming 2	−0.98	0.03
ENTPD4	ectonucleoside triphosphate diphosphohydrolase 4	1.02	0.0197
GBA	glucosidase, beta, acid	0.79	0.0264
IGF2R	insulin-like growth factor 2 receptor	1.11	0.0425
KIF11	kinesin family member 11	−0.82	0.0455
KIF20A	kinesin family member 20A	−1.12	0.0322
KIF23	kinesin family member 23	−1.22	0.0057
KIF4A	kinesin family member 4A	−1.74	0.0157
LIPA	lipase A, lysosomal acid, cholesterol esterase	−0.97	0.041
MCOLN1	mucolipin 1	0.88	0.0412
NDC80	NDC80 kinetochore complex component	−1.71	0.006
NUSAP1	nucleolar and spindle associated protein 1	−1.16	0.0229
PLK1	polo-like kinase 1	−1.42	0.0049
PPT2	palmitoyl-protein thioesterase 2	1.08	0.0072
PRC1	protein regulator of cytokinesis 1	−1.37	0.0049
RCC2	regulator of chromosome condensation 2	0.99	0.0234
SKA2	spindle and kinetochore associated complex subunit 2	−1.36	0.0231
SLC11A2	solute carrier family 11 member 2	1.44	0.0415
SORT1	sortilin 1	1.66	0.0007
STIL	SCL/TAL1 interrupting locus	−1.28	0.0156
TPX2	TPX2, microtubule-associated	−1.28	0.007
TTK	TTK protein kinase	−1.59	0.0008
ZWILCH	zwilch kinetochore protein	−0.77	0.0497

DRGs between VECs cultured in CM and CTR implicated in the enriched pathways. In the table are listed the genes involved in the significant enriched pathways from BioPlanet, GO Biological Process, and KEGG on the Enrichr website by uploading DRGs obtained by Transcriptome Analysis Console (TAC). Fold changes (FC) and adjusted *p*-values (FDR *p*-value) are depicted in the third and fourth column, respectively.

**Table 7:** Pathway enrichment analysis (Enrichr) of DRGs between LECs cultured in CM and the relative CTR.

Pathway	Overlap	Adj. *p*-Value
**BioPlanet**		
Translation	117/151	9.03 × 10^−30^
Cytoplasmic ribosomal proteins	87/108	1.74 × 10^−24^
Gene expression	424/968	1.79 × 10^−17^
Parkinson’s disease	87/131	2.14 × 10^−15^
Cell cycle	222/453	4.52 × 10^−15^
Oxidative phosphorylation	86/136	2.42 × 10^−13^
Proteasome degradation	49/63	1.06 × 10^−12^
DNA replication	116/207	1.19 × 10^−12^
Alzheimer’s disease	99/169	2.03 × 10^−12^
Huntington’s disease	105/184	3.54 × 10^−12^
Antigen processing: cross presentation	52/79	4.99 × 10^−9^
T-cell receptor regulation of apoptosis	255/603	1.25 × 10^−8^
S phase	65/112	5.41 × 10^−8^
Apoptosis regulation	49/78	1.41 × 10^−7^
Antigen presentation: folding, assembly, and peptide loading of class I MHC proteins	120/255	5.27 × 10^−7^
DNA replication pre-initiation	52/88	7.94 × 10^−7^
Mitotic G1-G1/S phases	72/135	7.94 × 10^−7^
Cell cycle checkpoints	64/117	1.26 × 10^−6^
Messenger RNA processing	97/203	4.43 × 10^−6^
Lysosome	63/121	1.55 × 10^−5^
Translation factors	32/50	2.82 × 10^−5^
M-phase pathway	52/96	2.99 × 10^−5^
Protein processing in the endoplasmic reticulum	80/166	3.02 × 10^−5^
Proteasome complex	19/24	3.22 × 10^−5^
Immune system signaling by interferons, interleukins, prolactin, and growth hormones	122/280	5.09 × 10^−5^
Adherens junction cell adhesion	42/74	5.82 × 10^−5^
Spliceosome	63/127	1.07 × 10^−4^
Apoptosis	104/242	4.55 × 10^−4^
Mitotic G2-G2/M phases	45/87	5.31 × 10^−4^
Cholesterol biosynthesis	17/24	9.85 × 10^−4^
Renal cell carcinoma	37/70	0.001303568
Transcription	79/181	0.001762513
Ubiquitin-mediated proteolysis	62/136	0.002129516
Pathogenic Escherichia coli infection	30/57	0.005652786
N-glycan biosynthesis	26/48	0.007204858
Messenger RNA splicing: major pathway	34/68	0.007574914
Glutathione metabolism	27/51	0.008633558
Colorectal cancer	31/62	0.011858167
Interleukin-2 signaling pathway	299/847	0.01193764
Focal adhesion	93/233	0.014128026
Mitotic prometaphase	23/43	0.01576878
Valine, leucine, and isoleucine degradation	23/44	0.021845628
Cellular response to hypoxia	15/25	0.022274613
Pancreatic cancer	33/70	0.024629677
Neurophilin interactions with VEGF and VEGF receptor	05/05	0.02670186
Endocytosis	80/201	0.02766493
Cell cycle progression regulation by PLK3	12/19	0.03136828
Phagosome	63/154	0.033723598
Meiosis	37/83	0.041409388
COPII-mediated vesicle transport	07/09	0.043591541
Peroxisome	35/78	0.043974351
Antigen processing and presentation	36/81	0.046768701

Pathway enrichment analysis of DRGs between LECs cultured in CM. Analysis was performed comparing the BioPlanet, GO Biological Process, and KEGG on the Enrichr website by uploading DRGs obtained by Transcriptome Analysis Console (TAC). In the table are listed the number of regulated genes compared with total number of genes in the pathway (second column) and *p*-value adjusted for multiple testing (last column).

**Table 8:** List of genes and the enriched pathways associated with metabolic mechanisms differentially modulated in LECs and VECs.

Gene Symbol	Description	Log2 FC		FDR *p*-Value	
		LECs	VECs	LECs	VECs
**Glycolysis**					
GAPDH	glyceraldehyde-3-phosphate dehydrogenase	−0.04	0.14	0.9046	0.7075
PGK1	phosphoglycerate kinase 1	−2.47	0.12	3.22 × 10^−7^	0.6417
PKM	pyruvate kinase, muscle	−2.18	0.44	1.81 × 10^−7^	0.2076
ENO1	enolase 1	−0.41	0.14	0.1924	0.7325
**One-carbon metabolism/nucleotide synthesis**					
PHGDH	phosphoglycerate dehydrogenase	1.5	1.04	1.88 × 10^−5^	0.007
SHMT2	serine hydroxymethyltransferase 2	0.84	0	0.0719	0.5407
CAD	carbamoyl-phosphate synthetase 2, aspartate transcarbamylase, and dihydroorotase	0.09	−0.12	0.7939	0.8057
IMPDH2	IMPdehydrogenase 2	−2.13	0.53	1.19 × 10^−6^	0.4923
MTHFD2	methylenetetrahydrofolate dehydrogenase (NADP+ dependent) 2	4.45	0.57	1.29 × 10^−9^	0.3519
NME4	NME/NM23 nucleoside diphosphate kinase 4	−1.86	0.01	1.44 × 10^−6^	0.9645
NME1-NME2; NME1; NME2	NME1-NME2 readthrough; NME/NM23 nucleoside diphosphate kinase 1; NME/NM23 nucleoside diphosphate kinase 2	−2.6	−0.26	4.29 × 10^−8^	0.8329
TYMS	thymidylate synthetase	−2.05	−0.85	1.46 × 10^−5^	0.2409
**Urea/TCA cycle**					
ODC1; SNORA80B	ornithine decarboxylase 1; small nucleolar RNA, H/ACA box 80B	−0.98	−0.5	0.0031	0.2514
SRM	spermidine synthase	−2.93	0.62	2.63 × 10^−8^	0.2849
ASS1	argininosuccinate synthase 1	−0.065	−0.44	0.0653	0.3289
PYCR1	pyrroline-5-carboxylate reductase 1	−0.57	0.28	0.2041	0.6933
ASNS	asparagine synthetase	6.03	1.51	4.11 × 10^−12^	0.0008
GOT1	glutamic-oxaloacetic transaminase 1	0.83	−0.09	0.0125	0.8934
**Oxidative Phosphorylation Genes**					
COX7C	cytochrome c oxidase subunit VIIc	−3.24	0.15	5.83 × 10^−10^	0.8302
COX5B	cytochrome c oxidase subunit Vb	−3.06	0.26	1.84 × 10^−8^	0.5633
NDUFB7	NADH dehydrogenase (ubiquinone) 1 beta subcomplex, 7	−0.98	−0.17	0.0177	0.9995
COX7B	cytochrome c oxidase subunit VIIb	−5.03	−0.27	2.98 × 10^−9^	0.8898
ATP5G1	ATP synthase, H+ transporting, mitochondrial Fo complex subunit C1	−4.59	−0.88	8.96 × 10^−8^	0.1972
COX6B1	cytochrome c oxidase subunit VIb polypeptide 1	−2.01	−0.43	3.26 × 10^−7^	0.3624
ATP5D	ATP synthase, H+ transporting, mitochondrial F1 complex, delta subunit	−4.36	0.93	2.42 × 10^−9^	0.0774
NDUFB6	NADH dehydrogenase (ubiquinone) 1 beta subcomplex, 6	−1.55	−0.59	0.0003	0.2409
NDUFS3	NADH dehydrogenase (ubiquinone) Fe-S protein 3	−0.12	0.06	0.7068	0.6352
NDUFS2	NADH dehydrogenase (ubiquinone) Fe-S protein 2	−1.91	0.23	0.0006	0.8406
NDUFS1	NADH dehydrogenase (ubiquinone) Fe-S protein 1	−0.15	0.15	0.6976	0.78
UQCRH	ubiquinol-cytochrome c reductase hinge protein	−2.62	−0.04	7.26 × 10^−8^	0.8027
UQCRFS1	ubiquinol-cytochrome c reductase, Rieske iron-sulfur polypeptide 1	−2.06	−0.05	2.23 × 10^−5^	0.6411
NDUFA7	NADH dehydrogenase (ubiquinone) 1 alpha subcomplex, 7	−2.98	−0.03	8.83 × 10^−8^	0.9319
ATP6V0C	ATPase, H+ transporting, V0 subunit c	−0.33	0.36	0.3446	0.2515
UQCR10	ubiquinol-cytochrome c reductase, complex III subunit X	−3.1	−0.53	4.90 × 10^−9^	0.165
ATP5A1	ATP synthase, H+ transporting, alpha subunit 1	−3.07	−0.02	1.84 × 10^−8^	0.6776
ATP5B	ATP synthase, H+ transporting, beta polypeptide	−1.54	0.3	0.0002	0.5228
NDUFA8	NADH dehydrogenase (ubiquinone) 1 alpha subcomplex, 8	−0.5	0.63	0.2039	0.1373
COX5A	cytochrome c oxidase subunit V a	−2.88	−0.19	2.15 × 10^−9^	0.7889
ATP6AP1	ATPase, H+ transporting, lysosomal accessory protein 1	−1.65	0.67	1.12 × 10^−5^	0.0482
SDHD	succinate dehydrogenase complex subunit D	−2.96	−0.11	5.72 × 10^−9^	0.9527
NDUFV1	NADH dehydrogenase (ubiquinone) flavoprotein 1	−1.15	−0.08	0.0021	0.9782
COX8A	cytochrome c oxidase subunit VIIIA	−5.88	0.29	1.82 × 10^−10^	0.8555
UQCRC1	ubiquinol-cytochrome c reductase core protein I	−1.26	−0.06	0.0004	0.9944
ATP5J2	ATP synthase, H+ transporting, mitochondrial Fo complex subunit F2	−4.11	−0.16	1.71 × 10^−10^	0.7345
COX7A2	cytochrome c oxidase subunit VII a polypeptide 2	−2.15	0.01	6.82 × 10^−6^	0.6142
COX4I1	cytochrome c oxidase subunit IV isoform 1	−1.3	−0.09	0.0001	0.7254
UQCRB	ubiquinol-cytochrome c reductase binding protein	−0.73	0.01	0.0115	0.9482
ATP5C1	ATP synthase, H+ transporting, gamma polypeptide 1	−2.13	−0.11	1.84 × 10^−6^	0.5134
ATP5G3	ATP synthase, H+ transporting, subunit C3	−1.67	−0.43	1.02 × 10^−5^	0.3869
ATP5F1	ATP synthase, H+ transporting, subunit B1	−2.3	−0.05	8.61 × 10^−8^	0.7665
COX6A1	cytochrome c oxidase subunit VI a polypeptide 1	−3.53	−0.02	6.41 × 10^−10^	0.9707

Pro- and anti-angiogenesis regulators in VECs and LECs cultured in CM and CTR. Log2 Fold changes (FC) and adjusted *p*-values (FDR *p*-value) of VECs and VECs are depicted in the table.

## Data Availability

All data supporting the findings of this study are available within the article and its supplemental data file or from the corresponding author upon reasonable request.

## Supplementary Materials

The following supporting information can be downloaded at: https://www.mdpi.com/article/10.3390/ijms23137192/s1.
