# Supplementary material for: Transcriptomic and Functional Evidence for Differential Effects of MCF-7 Breast Cancer Cell-Secretome on Vascular and Lymphatic Endothelial Cell Growth
Source: Int J Mol Sci. 2022 Jun 28;23(13):7192. doi: 10.3390/ijms23137192 (PMC9266834; doi:10.3390/ijms23137192)
Supplement: Supplementary file 1 [file ijms-23-07192-s001.zip › Supplementary data S1.pdf]

Article

Transcriptomic and Functional Evidence for Differential Effects of MCF-7 Breast Cancer Cell-Secretome on Vascular and Lymphatic Endothelial Cell Growth

Giovanna Azzarito [1], Michele Visentin [2], Brigitte Leeners [1], and Raghvendra K. Dubey [1,3]

[1] Department of Reproductive Endocrinology, University Hospital Zurich, 8952 Schlieren, CH;
giovanna.azzarito@usz.ch , Brigitte.leeners@usz.ch , raghvendra.dubey@usz.ch

[2] Department of Clinical Pharmacology and Toxicology, University Hospital Zurich, University of Zurich, Zurich, Switzerland; Michele.Visentin@usz.ch

[3] Department of Pharmacology & Chemical Biology, University of Pittsburgh, Pittsburgh, PA 15219, USA

Supplementary data S1

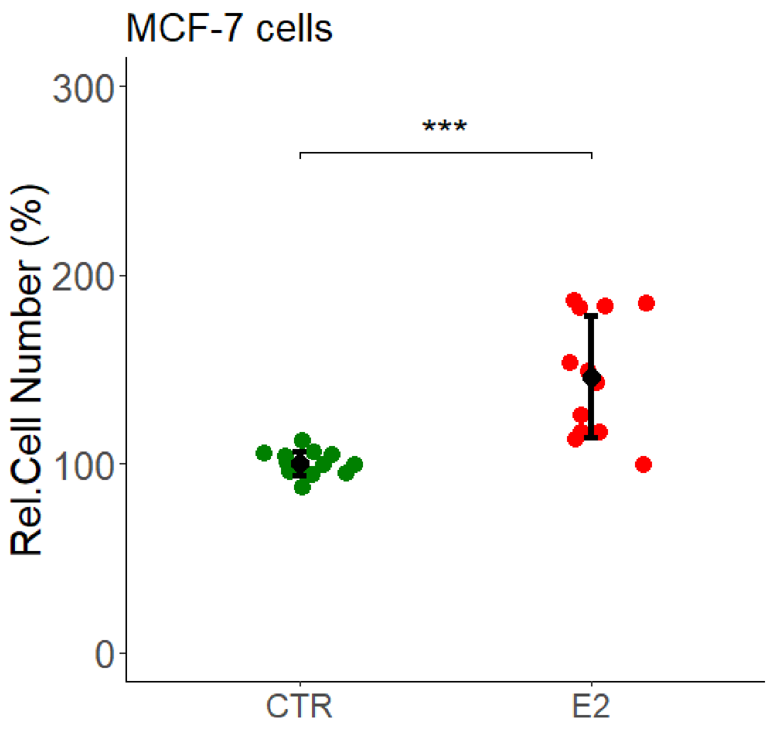

Figure S1: **Depicting the proliferative actions of estradiol (10nM) on MCF-7 breast cancer epithelial cells.** Experiments were performed at least 3 times in triplicates or quadruplicates and the data represent mean ± SD. p<0.01***, compared to the respective control.
